# Effect of Polydextrose and Its Complex on the Pasting, Function, and Microstructural Characteristics of Low-Gluten Wheat Dough and Steamed Buns

**DOI:** 10.3390/gels12090764

**Published:** 2026-08-26

**Authors:** Shiyu Chang, Ruijun Chen, Bing Dai, Xingjun Li

**Affiliations:** 1 Academy of National Food and Strategic Reserves Administration, National Engineering Research Center for Grain Storage and Transportation, Beijing 102209, China; csy@ags.ac.cn (S.C.); ruijunchen1@126.com (R.C.); bingdai3@126.com (B.D.); 2 College of Food Science and Engineering, Wuhan Polytechnic University, Wuhan 430048, China

**Keywords:** steamed bun, polydextrose, fructooligosaccharides, dietary fiber-rich food, air bubble cells, specific volume

## Abstract

To improve the gluten strength of low-gluten dough and achieve better-quality steamed buns, this study analyzed the pasting, function, and microstructural characteristics of low-gluten wheat dough and steamed buns with added 3–10% polydextrose (PD) and a complex dietary fiber (PIF) (i.e., PD, inulin, and fructooligosaccharides). Compared to the control, adding 3–10% PD or PIF to low-gluten flour increased the texture parameters of steamed buns, including the hardness, adhesiveness, cohesiveness, gumminess, and chewiness, with maximal improvements at 5% PD or PIF addition, while springiness and resilience decreased. Further, the surface color and brightness increased while the color difference decreased. The sensory parameters, including the specific volume, stickiness, toughness, and elasticity, improved, leading to an increased total score. Adding 3–10% PD or PIF reduced the retrogradation viscosity, retrogradation torque, and retrogradation of amylopectin in low-gluten flour. The dough development time increased with increasing dietary fiber (DF), consistent with the better adhesiveness and elasticity in the sensory analysis. As the DF addition amount increased, the breakdown viscosity of flour suspensions and the breakdown torque of the dough decreased. However, the pasting temperature of flour suspensions and the peak temperature of gelatinization both increased. The percentages of α-helices, β-turns, and random coils increased with increasing PD or PIF addition in both dough and steamed buns, indicating increasing stability of gluten proteins. Microstructural observations confirmed that, compared to added PD, the pores and interspaces in the dough became more numerous, larger, and deeper with increasing PIF addition from 3% to 10%. Similar to PD addition, as the PIF addition increased from 3% to 10%, the resistance to gluten network formation increased, the number of air bubble cells in steamed buns gradually decreased, and the chewiness increased; however, compared to PD addition, PIF addition resulted in a larger specific volume of the steamed buns, greater adhesive force, and easy detachment of large crumbs from the cut surface, while the hardness and sensory scores of the steamed buns increased. This study could help develop DF-rich low-gluten steamed buns with better mouthfeel and textural parameters.

## 1. Introduction

The European Food Standards Agency (EFSA) recommends a dietary reference value for dietary fiber of 25 g per day for adults aged 18 years or over for the important purpose of maintaining normal abdominal function but notes that higher levels of consumption are much more beneficial [1]. The National Health and Nutrition Examination Survey (NHANES) found that U.S. residents 20 years and older consume only 61% of the recommended value [2]. Severe dietary fiber (DF) deficiency is prevalent among Chinese residents, with an average daily intake of only 10–15 g/day, far below the recommended standard of 25–30 g/day in the “Dietary Guidelines for Chinese Residents (2022)” [3]. This lack of DF is a major root cause of the higher incidence of chronic diseases such as obesity, diabetes, cardiovascular disease, and colorectal cancer [4]. Whole grains and soluble DF are important means of supplementing the daily DF required by the human body [5]. However, whole grains are limited by their poor taste, texture, and storability. By contrast, the production and usage of soluble DF, such as polydextrose (PD), inulin, and fructooligosaccharides (FOS), have developed rapidly in recent years.

Polydextrose is widely recognized for its amorphous nature, high solubility, and favorable thermal stability, making it attractive as a low-calorie carrier in the food industry [6]. China produced 101,000 tons of PD in 2020; production is expected to reach 135,000 tons in 2026 [7]. The use of PD in health foods and baked foods accounts for 39% and 11.8% of the total use of PD in China, respectively [7]. However, the addition of PD and its complexes in staple cereal foods like steamed buns is limited.

Steamed buns, made via steaming fermented wheat dough, have a history of more than 1700 years and are a popular staple food in China and many Asian countries [8]. The gluten strength and water absorption rate of wheat flour directly affect the extensibility of the dough and the softness of steamed buns; high-quality flour ensures a stable structure and a delicate texture for steamed buns [9]. PD has excellent water-binding capabilities that help stabilize the gluten fraction [10] in bread dough [11] during freezing and thawing processes. Jing et al. [12] reported that 1–9% addition of PD as a soluble fiber had little influence on the sensory properties of steamed buns made with medium-gluten flour, with a total sensory score in the range of 87–90 (on a scale of 100). However, the hardness of steamed buns reached a maximum of 562.7 ± 69.7 g at 3% PD addition and was near the level of the control (no PD addition) at 7% PD addition. Liu et al. [13] added 3–10% PD to steamed buns made with high-gluten flour and found that PD addition maintained the total sensory score in the range of 67.6–69.1, while 5–7% PD addition significantly increased the hardness of steamed buns, with a maximum of 1394 ± 71 g. By contrast, 3% addition resulted in similar hardness to control buns. Given that steamed buns made with different gluten flours have different hardness/softness and sensory scores, it is worth exploring the potential effects of the addition of PD and its complexes on the textural, thermal, and functional properties of steamed buns made with low-gluten wheat flour.

Low-gluten flour is a type of wheat flour with a low protein content and low gluten strength, as well as good extensibility; it is suitable for making cookies, pastries, and other foods [14]. The wet gluten content of low-gluten flour must be less than 26% [15]. China’s annual output of low-gluten wheat is approximately 300,000–350,000 tons. Xiong et al. [14] reported that 2.5–7.5% inulin addition increased the gluten strength of low-gluten flour. The increased gluten strength had a considerably positive impact on food processing and the quality of the final product [14]. In the present study, it was hypothesized that strongly hygroscopic, amorphous PD would compete for water molecules during steaming. In the context of low-gluten, high-starch flour dough, this would result in a better sensory evaluation score for steamed buns. In addition, PD plays a role in controlling the appetite and blood sugar, while inulin, as a natural prebiotic, has significant effects on the gut microbiota and mineral absorption, and fructooligosaccharides can improve the intestinal micro-ecology and reduce the risk of cardiovascular disease [16,17,18,19,20]. Thus, the processed product from three water-soluble DFs (34% *w*/*w* PD, 33% *w*/*w* inulin, and 33% *w*/*w* fructooligosaccharides) was added to low-gluten wheat flour, and their effects on the thermo-mechanical properties of dough and the textural profiles of cooked steamed buns were observed. The purpose of this study is to improve the chewiness and DF content of steamed buns made with low-gluten wheat flour through the addition of PD and its complex, thereby preventing and alleviating the incidence of noninfectious chronic diseases in residents who mainly eat steamed buns.

## 2. Results and Discussion

### 2.1. Effect of Added DF on the Appearance and Sensory Attributes of Steamed Buns

The recommended daily dietary fiber intake for Chinese adults is 25–30 g, but more than 60% of the domestic population consumes less than the recommended amount [4]. Regular low-gluten wheat flour contains 1–2% DF [21]. Thus, we tested adding 3–10% PD or PIF into steamed buns made with low-gluten flour, because the addition of more than 10% DF can severely affect the speed of bread fermentation [22]. The effect of adding different amounts of PD or PIF on the specific volume of steamed buns is shown in Table S1. As for the statistical analysis of the dietary fiber addition effect, univariate–general linear model (UGLM) analysis in SPSS software is well-suited for testing the influence of unequivocal independent variables on continuous dependent variables, encompassing main effects, interaction effects, and covariate analysis [23]. Considering the DF species (PD and PIF) and addition amounts (0%, 3%, 5%, 7%, and 10%), we analyzed the main effects of adding DF on the specific volume of steamed buns (Table 1).

When compared to steamed buns made with high-gluten flour [13], steamed buns made with low-gluten flour had a lower specific volume.

**Figure 1:**
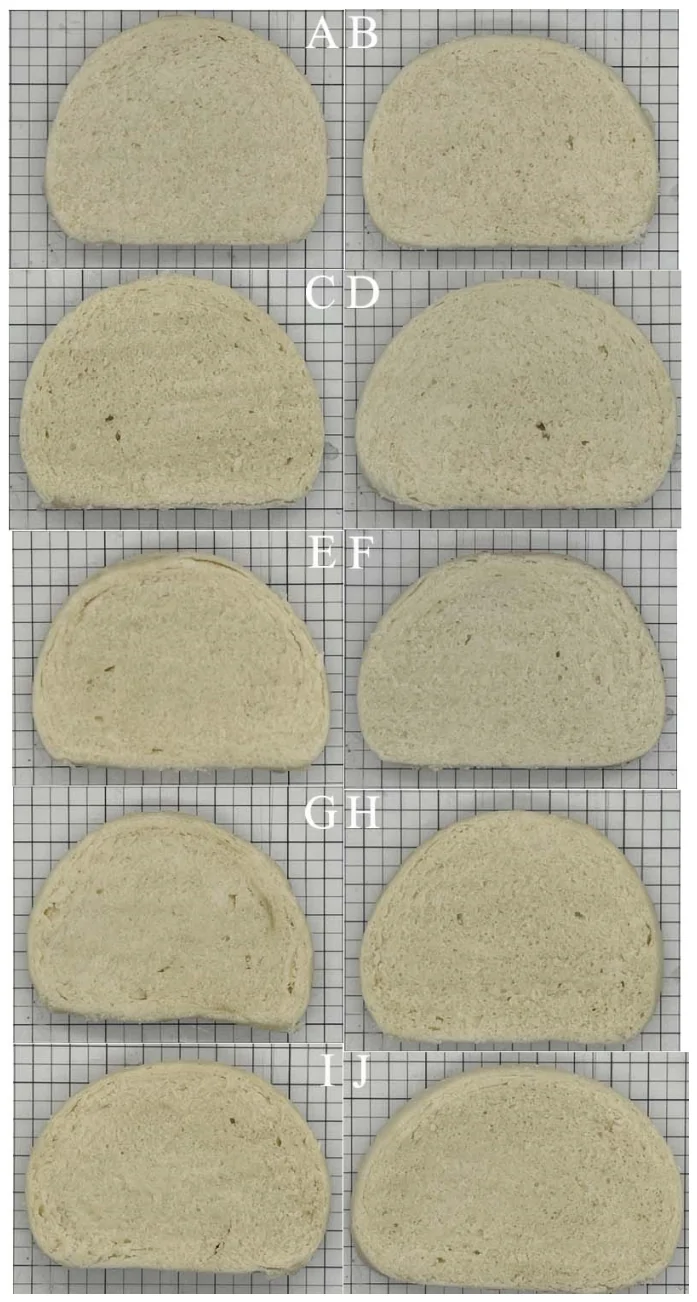
Effect of PD or PIF on the appearance of steamed buns made with low-gluten wheat. Notes: (**A**,**B**) are the control samples; (**C**,**E**,**G**,**I**) are the samples with added 3%, 5%, 7% and 10% PD, respectively; (**D**,**F**,**H**,**J**) are the samples with added 3%, 5%, 7% and 10% PIF, respectively. All photos are enlarged 1×.

Compared to no DF addition, both PD and PIF additions increased the volume, mass, and specific volume of steamed buns made with low-gluten wheat flour, with PD addition producing a smaller increase in specific volume than PIF addition (Table 1). With an increase in the amount of added DF, the volume, mass, and specific volume of steamed buns tended to increase; 10% addition resulted in the maximum increase. These results are similar to the trial results of Jing et al. [12], where 1%, 5%, 7%, and 9% PD additions significantly raised the volume and specific volume of steamed buns processed using medium-gluten flour, and 3%, 7% and 9% PD additions increased the mass of the steamed buns. However, the present results are different from the trial results of Liu et al. [13], who found that 3–10% PD addition resulted in a smaller specific volume than control steamed buns made with high-gluten flour. A possible explanation is that with an increase in PD or PIF addition, a raw steamed bun with the same weight could absorb more vapor during the steaming process, yielding increases in the volume, weight, and specific volume of the steamed bun, and steamed buns made with low-gluten flour might absorb more water vapor than steamed buns made with high-gluten flour. This assertion is further supported by the cross-sections in Figure 1, where it can be seen that the steamed bun made with low-gluten flour had more pores as the amount of added DF increased, with PIF addition yielding a relatively greater volume than PD addition.

Compared to steamed buns made with high-gluten flour [13], steamed buns made with low-gluten flour had a higher brightness index, greenness, and yellowness, and a lower color difference (Table 2 and Table S2).

Compared to no DF addition, PD addition induced a greater brightness index for steamed buns than PIF addition, while there was a lower color difference than with PIF addition (Table 2). PD addition reduced the greenness and maintained the yellowness, but PIF addition maintained the greenness and reduced the yellowness. With an increase in DF addition from 3% to 10%, the brightness index for steamed buns continued to increase, and the color difference decreased. The maximum greenness and yellowness were observed with 10% and 7% addition, respectively.

A 3–10% PD addition increased the brightness index and color difference of steamed buns made with high-gluten flour in the study by Liu et al. [13], but in the current study, 3–10% PD or PIF addition increased the brightness index and decreased the color difference of steamed buns made with low-gluten flour. This might be because PD and the PD in PIF can refresh steamed buns, and low-gluten flour has a lower protein content [14]. Polydextrose might improve the water retention, elasticity, and tenderness (brightness) of steamed buns by forming a “hydration layer” that wraps around protein molecules and binds with the polar groups of proteins (such as —NH_2_ and —COOH) through hydrogen bonds [24].

Compared to steamed buns made with high-gluten flour [13], steamed buns made with low-gluten flour had higher scores for width/height ratio and internal structure, and similar scores for elasticity, surface color, surface structure, toughness, adhesiveness, taste, and total score (Table 3 and Table S3). This highlights the importance of the research and development of full-sensory digital and intelligent equipment.

Compared to no DF addition, both PD and PIF additions significantly increased the specific volume, elasticity, surface color, toughness, adhesiveness, and total score but reduced the width/height ratio (Table 3). PIF addition increased the surface and internal structure, but PD addition did not change these parameters; neither addition changed the taste value. PIF addition increased the total sensory score of steamed buns more than PD addition, possibly because PIF addition resulted in a greater specific volume, surface, and inner structure for steamed buns than PD addition.

The main disadvantages of using low-gluten wheat flour to make steamed buns are that the product has poor elasticity, a soft texture, and no chewiness [14]. Although PD and PIF additions could increase the elasticity and total sensory score in low-gluten steamed buns, textural measurements were further performed.

### 2.2. Effect of Added DF on the Texture Parameters of Steamed Bun

Compared to steamed buns made with high-gluten flour [13], steamed buns made with low-gluten flour had about 0.4 times the hardness, adhesive force, gumminess, and chewiness, two-thirds the adhesiveness, 0.83 times the cohesiveness, much lower springiness, and much larger resilience (Table 4 and Table S4).

Compared to no DF addition, the addition of PD and PIF both increased the hardness, adhesive force, adhesiveness, cohesiveness, gumminess, and chewiness and decreased the springiness and resilience (Table 4). The increasing effect of PIF addition on the hardness, adhesive force, and adhesiveness was greater than that of PD. The addition of PIF and PD resulted in similar springiness, cohesiveness, resilience, gumminess, and chewiness. With an increase in DF addition, the springiness and resilience of steamed buns continued to decrease, while the maximum hardness, adhesiveness, adhesive force, gumminess, cohesiveness, and chewiness were greatest around 5% addition.

The present study shows that adding 3–10% PD or PIF to low-gluten flour indeed increased the hardness, cohesiveness, gumminess, and chewiness of steamed buns, improving the textural parameters. Ansari et al. [25] showed that 10% PD or inulin addition maintained the specific volume and crumb hardness of soft wheat bread, while 10% PD addition increased the chewiness, aroma, taste, and total acceptability, but 10% inulin addition maintained these parameters. In the present study, 3–10% PD or PIF addition increased the specific volume, hardness, chewiness, and total sensory score of low-gluten wheat buns, possibly because polydextrose creates a mesh that could envelop the starch and other particles of the flour, increasing the cohesiveness of the dough, enhancing gas retention, and thickening the wall of the surrounding gas cells [26].

### 2.3. Effect of Added DF on the Pasting and Thermal Parameters of Low-Gluten Wheat Flour

Compared to steamed buns made with high-gluten flour [13], steamed buns made with low-gluten flour had greater peak, trough, breakdown, and final viscosities, greater pasting peak time and temperature, but lower setback viscosity (Table 5 and Table S5).

Compared to no DF addition, PD and PIF additions decreased the trough, peak, breakdown, final, and setback viscosities, as well as the peak time, but increased the pasting temperature (Table 5, Figure S1). PIF addition had a stronger decreasing effect on the peak, breakdown, and final viscosities and a greater increasing effect on the pasting temperature than PD addition. With an increase in the amount of added DF, the trough, peak, breakdown, final, and setback viscosities, as well as the peak time decreased, but the pasting temperature increased.

In the present study, although 3–10% PIF addition reduced the setback viscosity of low-gluten wheat flour, it increased the hardness of low-gluten steamed buns, suggesting that PD, inulin, and fructooligosaccharide (FOS) molecules might not reduce the hydrogen bonds formed by the hydroxyl groups between starch molecules and might not interfere with the formation of ordered structures in the starch retrograde process, thereby maintaining the hardness of low-gluten flour gels. These results are different from those observed with the addition of 1–5% inulin with different masses, which resulted in a significant reduction in the hardness of rice starch gels [27].

Compared to steamed buns made with high-gluten flour [13], steamed buns made with low-gluten flour had lower onset (*T*_o_), peak (*T*_p_), and conclusion (*T*_c_) temperatures and peak width of gelatinization, with similar values of enthalpy and peak height of gelatinization (Table 6 and Table S6).

Compared to no DF addition, both PD and PIF additions increased the enthalpy, *T*_p_, and peak width of gelatinization; PD addition did not change the *T*_o_ and *T*_c_ values, but PIF addition increased them (Table 6, Figure S2). The increasing effect of PD on the enthalpy of gelatinization was greater than that of PIF. The maximal *T*_o_ and *T*_p_ values were observed at 10% DF addition, while the maximal enthalpy, *T*_c_, peak width, and peak height were observed at 5% addition.

Compared to steamed buns made with high-gluten flour [13], steamed buns made with low-gluten flour had a greater peak temperature of gelatinization and a similar gelatinization enthalpy and amylopectin aging when measuring the retrogradation of gelatinized paste stored at 4 °C for 21 days (Table 7 and Table S7, Figure S2). When the gelatinized paste was stored at 4 °C for 21 days, compared to no DF addition, PD and PIF additions decreased the enthalpy, *T*_p_, peak height, and aging. PD addition had a stronger inhibitory effect on aging than PIF addition. With an increase the amount of added DF, the enthalpy, *T*_p_, peak height, and aging of gelatinized paste decreased.

PD is a recognized food stabilizer [28], but inulin and fructooligosaccharides (FOS) have not been characterized as food stabilizers. In the present study, similar to PD addition, PIF addition decreased the setback viscosity of pasting and amylopectin aging of low-gluten wheat paste but increased the hardness and chewiness of low-gluten steamed buns. These results suggest that inulin and fructooligosaccharides have texture-modifying properties, which can contribute to the stability of food products.

### 2.4. Effect of Added DF on the Thermo-Mechanical Parameters of Low-Gluten Wheat Dough

Compared to steamed buns made with high-gluten flour [13], steamed buns made with low-gluten flour had higher development time and stability time of the dough, greater maximum gelatinization torque (C3) and starch breakdown (C3–C4), and lower protein weakness (C3–C4) and starch setback torque (C5–C4) (Table 8 and Table S8).

Compared to no DF addition, both PD and PIF additions increased the dough development time (DDT), persistent viscosity (C4), and protein weakness degree (C1–Cs), but decreased the dough stability time (DST), maximum consistency (C1), minimum consistency (C2), starch breakdown torque (C3–C4), amylase activity (C3/C4), starch setback torque (C5–C4), and gelatinization speed (β), whilst the speed of heating (α) and enzymatic degradation (γ) were unchanged. PIF addition had a stronger increasing effect on C4 and decreasing effects on C2, C3–C4, C3/C4, C5–C4, and β than PD addition. PD addition increased the maximum gelatinization torque (C3) and setback end torque (C5), but PIF addition decreased C3 and did not change C5.

With an increase in the amount of added DF, DDT, C4, C5, and C1–Cs increased, α did not change, but DST, C1, C2, C3, C3/C4, C3–C4, C5–C4, and β decreased. A 3% DF addition resulted in the maximal γ; thereafter, γ decreased with an increase in the amount of added DF.

The DDT refers to the time elapsed from the addition of water until the consistency reaches its maximum value [29]. The dough formation process is a complex process; as gluten continuously forms, the viscoelasticity of the dough gradually increases. Under mechanical action, the formation of a stronger gluten network enhances dough viscosity, resulting in greater elasticity and a correspondingly longer development time. An inulin addition of 2.5–7.5% had little effect on the development time of low-gluten dough [14]. In the present study, 7–10% PD or PIF addition increased the development time of low-gluten dough, but 3–5% did not change the DDT. This suggests that 7–10% PD or PIF addition results in better gluten formation in low-gluten flour.

The stability time of the dough refers to its tolerance to mechanical mixing; the length of the stability time reflects the dough’s kneading tolerance, i.e., its resistance to degradation by shear forces. A longer stability time indicates better dough toughness and structural support strength of the end product [30]. A 2.5–7.5% inulin addition has been shown to increase the stability time of low-gluten dough [14]. In the present study, 3–5% PD or PIF addition did not change the stability time of low-gluten dough, but 7–10% decreased the DST. These results suggest that, although 7–10% PD or PIF addition reduced the toughness of low-gluten dough, they indeed increased the toughness of low-gluten steamed buns during the tasting evaluation. PD addition tended to improve this property, while PIF addition significantly increased the surface and inner structure of low-gluten steamed buns possibly due to the filler function of polydextrose and inulin.

The weakening degree represents the extent to which the gluten weakens after dough mixing; a smaller weakening degree indicates stronger gluten. A 2.5–7.5% inulin addition can reduce the weakening degree of low-gluten dough [14], but in the present study, 10% PD or PIF addition increased the weakening degree of low-gluten dough, whereas 3–7% did not change the weakening degree (C1–Cs). A 3–7% PD or PIF addition maintained the gluten strength of low-gluten flour dough. This may be because the intermolecular crosslinking of low-concentration PD, inulin, and FOS forms a network and simultaneously interacts with gluten proteins to form a more complex system, improving the gluten network structure [31] and enhancing the stability of the dough.

Ansari et al. [32] hypothesized that the dilution of wheat gluten via added dietary fiber leads to breaking of the starch–gluten network and a decrease in dough stability due to a decrease in protein content. We believe that the dilution of gluten by dietary fiber alone cannot account for all of the variations observed with the addition of dietary fiber to wheat flour because particular hydrocolloids and fibers indeed improved the rheological characteristics of wheat flour, like the water absorption rate, dough stability, and dough development time [33].

### 2.5. Effect of Added DF on the Starch Structure and Protein Secondary Structure of Low-Gluten Wheat Dough and Steamed Buns

Compared to no DF addition, PD addition raised the amorphous regions of starch in dough (R_1022/995_) but decreased the crystal regions of starch (R_1047/1022_) and the interaction between protein and starch (R_1068/1022_), while PIF addition reduced the amorphous regions of starch in dough but did not change the crystal regions of starch and the interaction between protein and starch (Table 9 and Table S9, Figure S3). With an increase in the amount of added DF in dough from 3% to 7%, the amorphous regions of starch were comparable to those in the control but significantly higher than those in the control at 10% DF. The crystal regions of starch and the interaction between starch and protein were lower than those in the control at 3–10% DF addition. These results suggest that PD addition could release more amorphous regions from starch granules in raw dough, producing a soft dough, while PIF maintained or even tended to increase the crystal regions of starch and the interaction between protein and starch, forming a stronger dough structure.

Compared to no DF addition in steamed buns, PD and PIF additions increased the amorphous regions of starch but decreased the crystal regions of starch and the interaction between protein and starch. PD addition had a similar effect on the amorphous regions and crystal regions of starch and a decreasing effect on the interaction between protein and starch, compared to PIF addition. Among 3–10% DF additions, 3% and 10% DF additions yielded the maximal amorphous regions of starch, while 7% DF addition yielded higher crystal regions of starch and a greater interaction between protein and starch in steamed buns. These results indicate that PD addition induced softer steamed buns than PIF addition.

A 3–10% PD or PIF addition induced more amorphous regions of starch, less crystalline regions of starch, and less interaction between starch and protein in low-gluten steamed buns. These results might be because soluble amorphous dietary fiber powders can enhance food stability by modulating water interactions, molecular mobility, and amorphous matrix formation, while simultaneously delivering health benefits [34,35].

Compared to no DF addition in raw dough, PD addition decreased the β-sheet content but increased the contents of random coils, α-helixes, and β-turns, while PIF addition did not change the contents of these protein secondary structures (Table 10 and Table S10). With an increase in the amount of added DF from 3% to 10%, the contents of these protein secondary structures in dough remained essentially unchanged.

Compared to no DF addition in steamed buns, PD and PIF additions decreased the content of β-sheets but increased the contents of α-helixes, random coils, and β-turns. Aside from the higher content of random coils induced by PD addition, PIF addition yielded similar contents of protein secondary structures as PD addition. As the amount of DF added to steamed buns increased from 3% to 10%, the minimal percentage of β-sheets and the maximal contents of α-helixes, random coils, and β-turns occurred at 5% DF addition.

For dough processed using freeze-dried gluten protein powder, the addition of 2–8% barley β-glucan resulted in increased contents of α-helixes and β-sheets with increasing β-glucan content, while the contents of β-turns and random coils decreased with increasing β-glucan content [36]. By contrast, in dough and steamed buns made with low-gluten flour in this study, the contents of α-helixes, β-turns, and random coils increased with increasing PD or PIF content from 0% to 5%; thereafter, they remained constant or decreased with an increase in the PD or PIF content from 7% to 10%, whereas the β-sheet content showed an opposite trend with increasing PD or PIF addition. The increase in α-helix content indicates a more uniform and compact internal structure of the gluten proteins. The difference in the trends for β-turns, β-sheets, and random coils may be attributed to the difference between pure gluten proteins and low-gluten flour ingredients. The added 3–5% PD or PIF might interact with gluten proteins via hydrogen bonds and hydrophobic interactions, thereby increasing the stability of gluten proteins.

### 2.6. Effect of Added DF on the Microstructure of Dough and Steamed Buns with Low-Gluten Wheat

**Figure 2:**
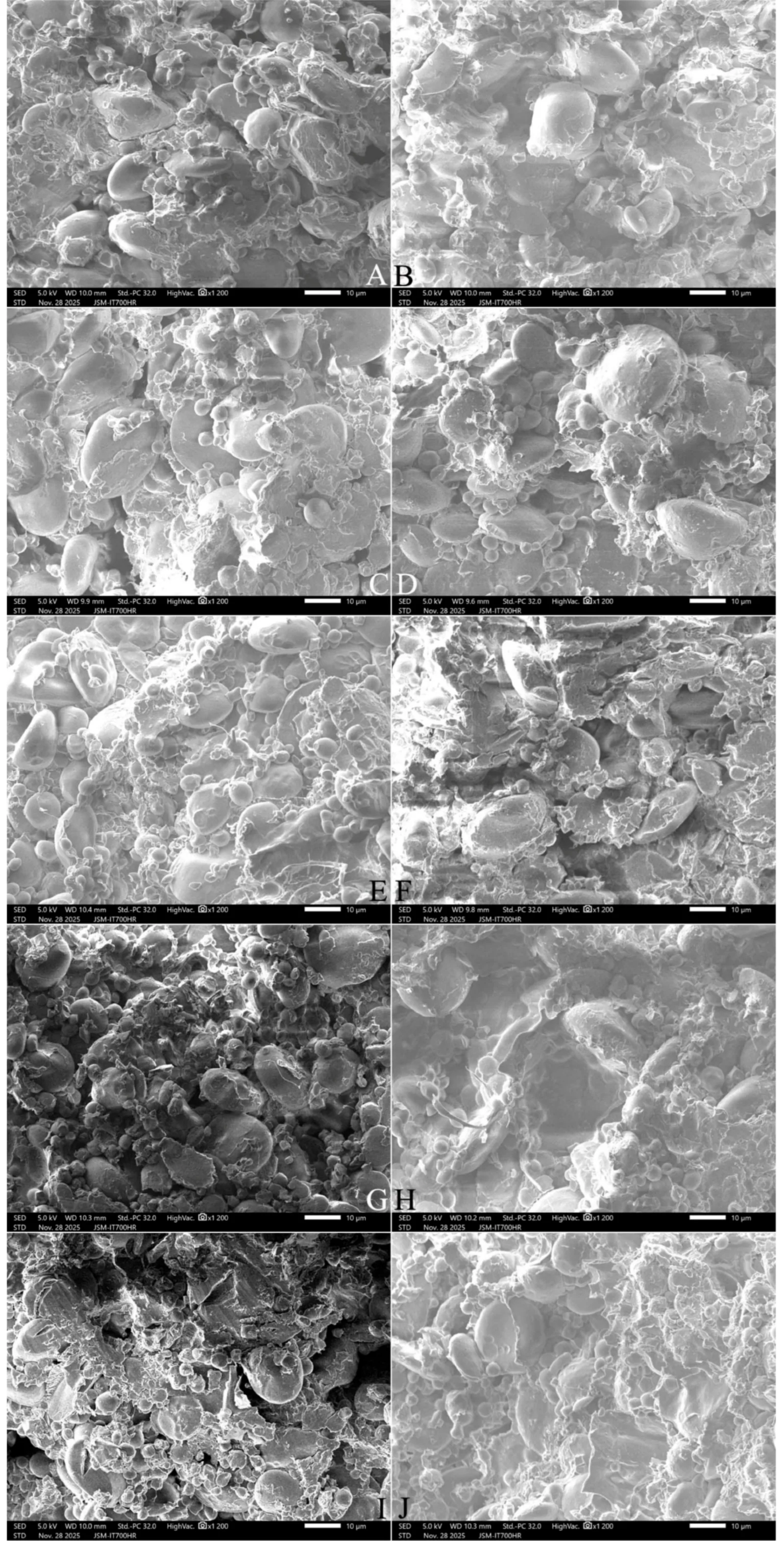
Effect of PD or PIF on the dough structure of low-gluten wheat. Notes: (**A**,**B**) are the control samples; (**C**,**E**,**G**,**I**) are the samples with added 3%, 5%, 7%, and 10% PD, respectively; (**D**,**F**,**H**,**J**) are the samples with added 3%, 5%, 7%, and 10% PIF, respectively. All photos are enlarged 1200×.

Figure 2 displays the microstructure of the unfermented dough at a magnification of 1200 times. In the control dough without PD, ellipsoidal or spherical starch granules were exposed at the surface, in the interspaces of the solidified gluten protein network, or firmly embedded into the gluten protein network, with obvious interstices between the starch granules and obvious boundaries (Figure 2A,B). Depending on their distinct structures, PDs can arrange a continuous three-dimensional network structure with some elasticity and adhesion. The addition of 3–7% PD had similar effects on the structure of the gluten network, slowly increasing the uniform pores and even the bubble-holding capacity of the dough (Figure 2C,E,G). At 10% added PD, the looseness and adhesion degree of the dough were further expanded, the holes were smaller and more numerous, and the borders between starch granules gradually were obscured (Figure 2I).

Compared to added PD, the pores and interspaces in the dough became more numerous, bigger, and deeper with increasing PIF addition from 3% to 10% (Figure 2D,F,H,J). At 7–10% added PIF, the largest holes were observed (Figure 2H,J). This may be because the similar proportions of PD, inulin, and fructooligosaccharides in PIF competed more strongly with the gluten and starch for water molecules and absorbed more water; thus, larger holes were formed upon freeze-drying the dough.

**Figure 3:**
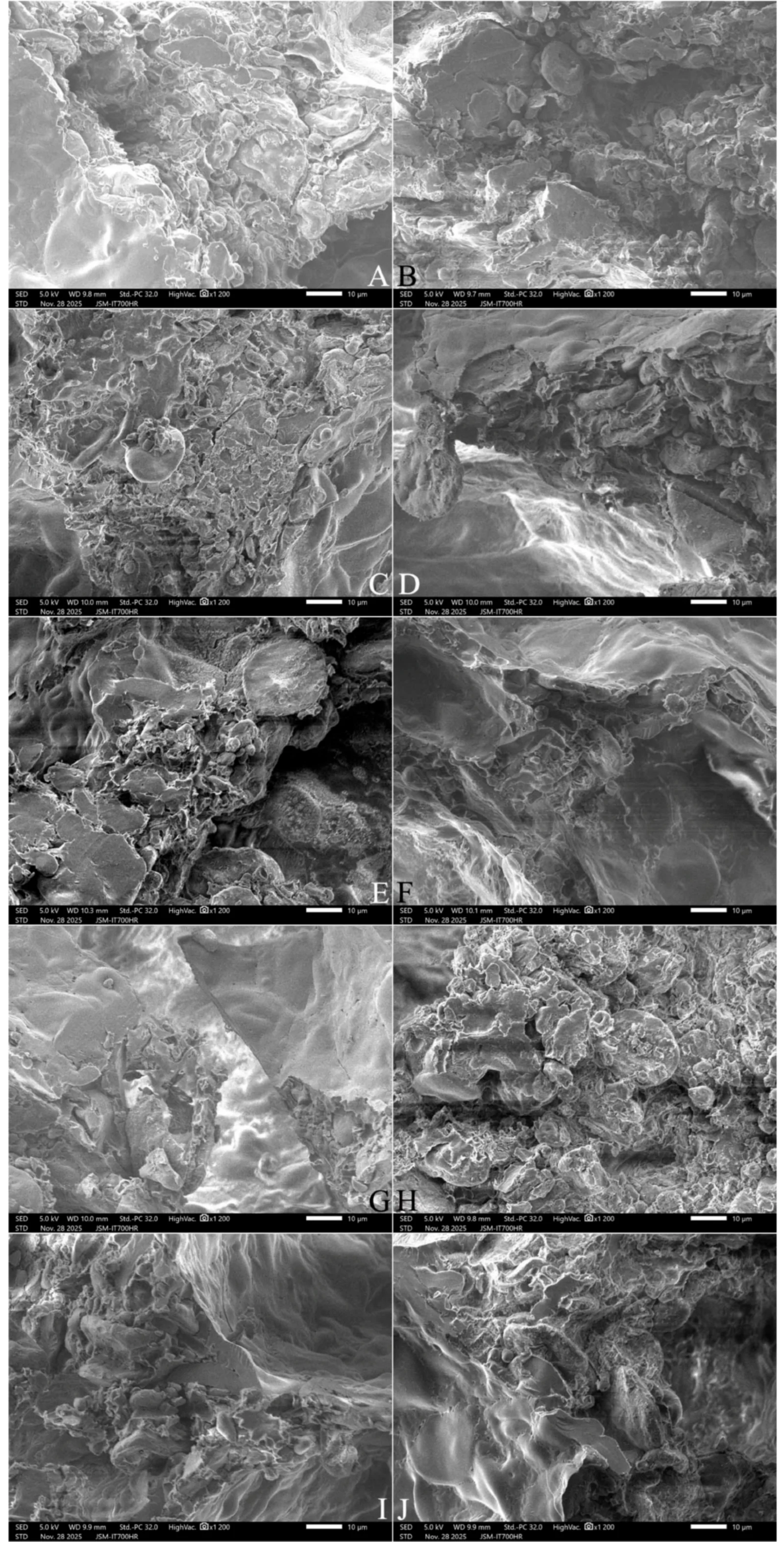
Effect of PD or PIF on the structure of steamed buns made with low-gluten wheat. Notes: (**A**,**B**) are the control samples; (**C**,**E**,**G**,**I**) are the samples with added 3%, 5%, 7%, and 10% PD, respectively; (**D**,**F**,**H**,**J**) are the samples with added 3%, 5%, 7%, and 10% PIF, respectively. All photos are enlarged 1200×.

Figure 3 displays the microstructure of a steamed bun at an enlargement of 1200 times. Compared to the control without PD (Figure 3A,B), the addition of 3% PD (Figure 3C) resulted in more and larger air bubble cells in the cross-section of the steamed bun and improved the uniformity of the gluten network structure. With the addition of 5–10% PD (Figure 3E,G,I), although the formation of the gluten network was increasingly affected, the gluten structure strength increased, the number of air bubble cells in the bun decreased, and the hardness increased, leading to the detachment of large crumbs from the cross-section and an uneven surface. However, during the steaming process of raw buns, the hygroscopicity of PD increased the amount of water vapor absorbed, resulting in an increased specific adhesiveness, reduced detachment of large crumbs, and increased chewiness and sensory scores for steamed buns.

Similarly, when PIF addition increased from 3% to 10% (Figure 3D,F,H,J), the resistance to gluten network formation increased, the number of air bubble cells in the bun gradually decreased, and the hardness increased. The hygroscopicity of PIF might be greater than that of PD, leading to a larger specific volume, greater adhesiveness, and easy detachment of large crumbs, resulting in increased chewiness and sensory scores for steamed buns.

The low viscosity of PD in solution endows it with humectant properties [37], allowing it to control unfavorable changes in the food moisture content by retaining moisture or preventing moisture migration. This inhibited the retrogradation of amylose and amylopectin in low-gluten steamed buns and extended the shelf life in the present study. A 10% PD or PIF addition also reduced gluten formation in low-gluten dough (Table 8), maintaining a flaky structure (Figure 2I,J and Figure 3I,J). The water solubility of inulin and fructooligosaccharides confers them with good flowability and dispersibility in food processes [31]. The addition of PD or a mixture of PD with inulin and fructooligosaccharides (PIF) to low-gluten steamed buns resulted in a fine and soft structure with distinct pores (Figure 3C–J).

## 3. Conclusions

Compared to previous research adding PD in medium- or high-gluten steamed buns, this paper is the first to examine the maintenance of gluten strength in low-gluten dough through PD or PIF addition. Compared to the control, adding 3–10% PD or PIF to low-gluten flour increased the textural and sensory parameters of steamed buns, including the hardness, adhesiveness, cohesiveness, gumminess, and chewiness, while enhancing the brightness, specific volume, toughness, stickiness, and total sensory score, but inhibited the retrogradation of amylose and amylopectin, suggesting that the shelf life of the product could be extended. The Mixolab analysis showed that adding 3–7% PD or PIF maintained protein weakness, but 10% addition significantly weakened the gluten network; both DF additions improved the mouthfeel, texture, and product stability of low-gluten steamed buns. Compared to PD addition, adding 3–10% PIF resulted in a gradual decrease in the number of air bubble cells in steamed buns, a larger specific volume, greater adhesiveness and hardness, and increased chewiness and sensory scores. Further work is needed to compare the water sorption isotherms and storage stability of low-gluten steamed buns with added PD or PIF. The findings of this study contribute to the development of dietary fiber-rich steamed buns and pastries.

## 4. Materials and Methods

### 4.1. Samples

Polydextrose (PD) and complex dietary fiber (PIF) were provided by Runloy Biotechnology (Shanghai) Co., Ltd., Shanghai, China. The PIF consisted of 34% (*w*/*w*) polydextrose, 33% (*w*/*w*) fructooligosaccharides, and 33% (*w*/*w*) inulin. After mixing these raw materials uniformly, a small amount of water vapor or alcohol was added as a binder for granulation. The composite dietary fiber is approximately spherical, and the small spheres bond and aggregate into larger spheres. Low-gluten wheat flour (Gold Dragonfish, Wilmar Shanghai Co., Ltd., Shanghai, China) with 13% protein content and 18.2% wet gluten content was purchased from a local supermarket. PD and PIF were added to wheat flour at mass ratios of 0%, 3%, 5%, 7% and 10%, separately, to obtain PD- or PIF-added samples.

### 4.2. Textural Characteristics of Steamed Wheat Buns

**Figure 4:**
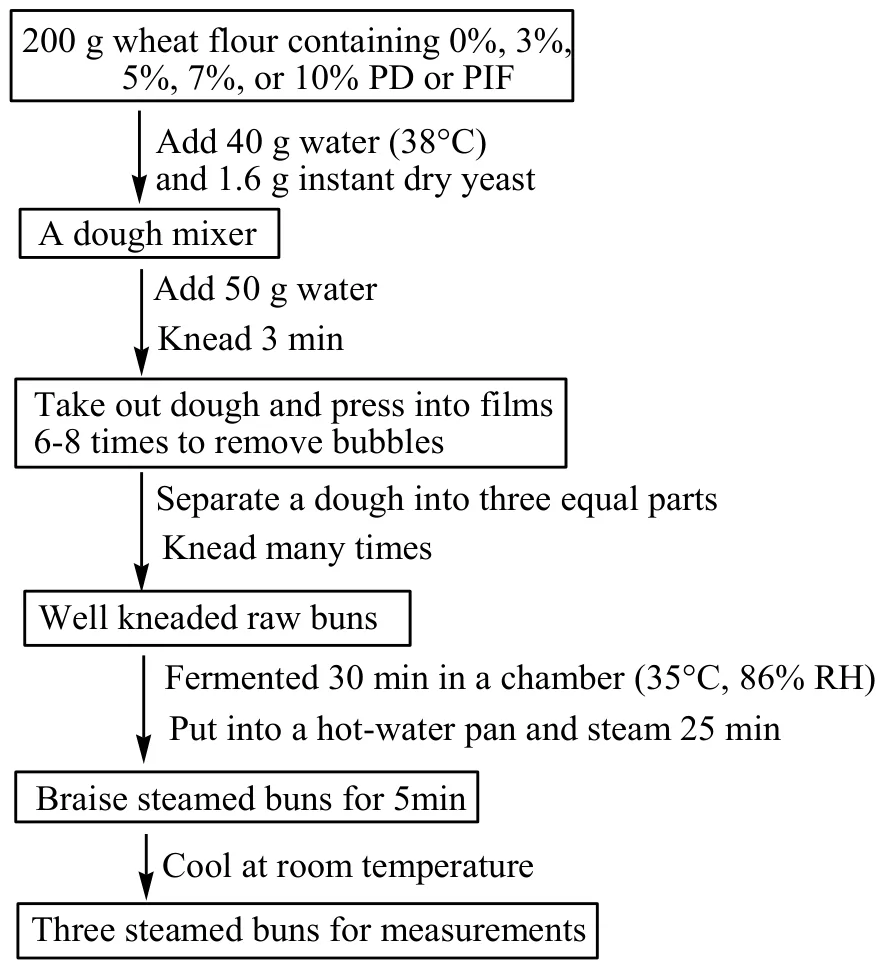
Process flow diagram for steamed buns in this study.

Steamed wheat buns were prepared according to the Chinese standard GB/T 35991-2018 [38]. First, 200 g of flour was weighed and mixed in a dough mixer (JHMZ 200, Dongfang Fude Technology Development Center, Beijing, China) with 40 g of 38 °C water containing 1.6 g of instant dry yeast (Angel Yeast Co. LTD., Yichang City, Hubei Province, China) (Figure 4). The dough was then kneaded for 3 min in the dough mixer at room temperature (RT) after adding 50 g of deionized water. Second, the dough was removed and pressed into disks 6–8 times using a rolling device (JCXZ, Dongfang Fude Technology Development Center, Beijing, China) to eliminate bubbles. Third, the dough was separated into three equivalent parts, and each part was kneaded again and folded toward the bottom edge to ensure that the front and back of the dough were kneaded the same number of times. Fourth, the well-kneaded raw buns were fermented for 30.0 min at 35 °C and 86% relative humidity (RH) in a fermentation box. Then, 1500 mL of water was added to a pan, and the buns were steamed for 25 min at 1600 W power. Finally, the steamed buns were left to rest for 5 min in the covered pan, then taken out and covered with two layers of gauze to cool at room temperature (RT) for 1 h prior to texture parameter measurements.

The textural profile of the steamed wheat buns was determined using a textural analyzer (CTX, Brookfield, Middleboro, MA, USA) equipped with Texture Pro 1.0.19 software, as described by Wang et al. [39]. A steamed bun was cut into cubes (10 mm × 10 mm × 10 mm) using a bread knife to obtain the test samples. The texture characteristics were measured using a cylindrical P35 probe (diameter of 3.5 cm, height of 4 cm). The parameters were set as follows: the test distance was a sample thickness of 50%, the speeds of the pre-test, test, and post-test were 1 mm/s, and the distance of compression was 12 mm, with a sensing force of 5 g, and the waiting time between two compressions was 13 s. The hardness (g), adhesiveness (mJ), adhesive force (g), cohesiveness, springiness (mm), resilience, gumminess (hardness × cohesiveness, g), and chewiness (hardness × cohesiveness × springiness, mJ) were measured.

### 4.3. Specific Volume and Color of Steamed Buns

The specific volume of the steamed buns was accurately determined in accordance with GB/T 21118-2007 [40]. The mass of the steamed buns was measured after being cooled for 1 h. The volume of the steamed buns was measured using the rapeseed replacement method; the average value was obtained after three repetitions. The specific volume was calculated using the following formula:

(1) $$Specificvolume\left(\frac{{cm}^{3}}{g}\right)=\frac{The\;volume\;of\;a\;steamed\;bun\;({cm}^{3})}{The\;mass\;of\;a\;steamed\;bun\;(g)}$$

The color value of the steamed buns was measured using a colorimeter (CR-100, Japanese Konica Minolta, Ltd., Shanghai, China). A steamed bun was cut into slices with a thickness of 15.0 mm to obtain test samples. A standard white plate was used for colorimeter calibration. Second, the steamed bun slices were placed on the test port, fully covering it, and a test key was pressed. The sample was removed when the test obtained a set of color differences. The colorimeter determined the brightness (L), redness–greenness (a*), and yellowness–blueness (b*) values, utilizing the color difference between the standard white plate and the tested steamed bun slices. ΔE represents the total color difference.

The slices of steamed buns were photographed using a mobile phone (iPhone 17, 4.8 million pixels, Apple, Beijing, China).

### 4.4. Sensory Attributes of Steamed Wheat Buns

The steamed wheat buns were prepared according to the Chinese national standard GB/T 35991-2018 [38]. For each DF-added sample, nine steamed buns were prepared for one sensory evaluation trial. The sensory attributes and their respective scoring points included specific volume (g/mL, 20 points), width/height ratio (5 points), surface color (10 points), surface structure (10 points), internal structure (20 points), elasticity (10 points), toughness (10 points), viscosity (10 points), and taste (5 points), with a total score of 100. Eight trained evaluators (four males and four females) aged between 23 and 28 years observed, tasted, and estimated the steamed buns. Each evaluator had a steamed bun sample for each DF addition. The samples were blinded and randomized, and the trained panelists assessed them at 10-min intervals between sample evaluations in each group, with plain water provided for rinsing during the interval. The evaluator could not smoke or eat food one hour before tasting but could drink water. During the tasting process, each evaluator was in a normal physiological state and could not use cosmetics or products with a stronger odor. The sensory testing was performed one hour before a meal or two hours after a meal.

### 4.5. Pasting Characteristics

An RVA device (Perkone Scientific Equipment Co., Ltd., Hangzhou, China) was used to determine the pasting parameters of the samples in accordance with GB/T 24852-2010 [41]. During a measurement, the stirring paddle speed was set at 960 r/min for the initial 10 s, then decreased to 160 r/min within 20 s, and held at 180 r/min. The temperature was first set at 50 °C for 1 min, then raised to 95 °C within 3.71 min, held for 2.5 min, then lowered to 50 °C within 2.8 min, and again held for 2 min.

### 4.6. Thermal Properties

PD or PIF was uniformly mixed with wheat flour on a mass basis to achieve addition levels of 0% to 10%. About 5.0 mg of sample was employed to determine the gelatinization parameters employing a differential scanning calorimeter (DSC 214, Netzsch GmbH, Selb, Germany), according to the method described by Wang et al. [39]. After gelatinization, the retrograded samples were placed in 24-well plates with covers at 4 °C for 21 d and again measured for retrogradation. The trial results were evaluated using the same analysis software. The amylopectin aging of the retrograded flour paste was obtained using Equation (2):

(2) $$\begin{array}{l} Amylopectin\;ageing\left(\%\right)\\ =\left(Gelatinization\;enthalpy\;decided\;at\;day\;21\right)\times 100/(Gelatinization\;enthalpy\;decided\;at\;day\;0) \end{array}$$

### 4.7. Thermo-Mechanical Characteristics

A Mixolab device (Chopin Technologies, Tripette et Renaud, Paris, France) was used to analyze the thermal-mechanical characteristics of the dough, as reported by Wang et al. [39]. For assays under a constant hydration, about 46.3 g of wheat flour was placed in a Mixolab bowl. Subsequently, 75 g of dough with 62% water content (14% moisture basis) was used in the evaluation. The temperature of the water tank during the initial mixing process was set at 30 °C. The mixing rate was fixed at 80 rpm/min during the whole analysis. The initial mixing test was performed at 30 °C for 8 min. The temperature was then increased to 90 °C within 15 min at a rate of 4 °C/min. After 7 min at 90 °C, the temperature was decreased to 50 °C within 10 min at a rate of 4 °C/min, and maintained for 5 min. The total time of one assay was 45 minutes.

### 4.8. Fourier Transform Infrared Spectroscopy (FTIR)

The FTIR spectra of the dough and steamed bun samples were acquired using a Nicolet 6700 FTIR spectrometer (Thermo Fisher Scientific, Waltham, MA, USA). The adopted testing conditions included a spectral resolution of 4 cm^−1^ and 64 scans, achieving a verified signal-to-noise ratio at the 100% T-line from 4300 to 4400 cm^−1^. All measurements were depicted using OMNIC software (Version 9.0). Samples were well ground with KBr at a mass ratio of 100:1 and pressed into tablets. The scanning wavelength range was 400–4000 cm^−1^. The wavelength intensity ratios R_1022/995_ and R_1047/1022_ indicate the short-range molecular order of a starch granule, describing the amorphous and crystalline regions of starch, respectively. R_1068/1022_ indicates the interaction between protein and starch [42]. The secondary protein structures and internal hydrogen bonding characteristics were analyzed using the spectral alterations in the amide I region (1600–1700 cm^−1^). The important protein secondary structure forms in this amide I region include α-helixes (1650–1660 cm^−1^), random coils (1640–1650 cm^−1^), β-sheets (1600–1640 cm^−1^), and β-turns (1660–1700 cm^−1^) [43].

### 4.9. Scanning Electron Microscopy (SEM)

The steamed wheat buns were cooled to room temperature, and small aliquots of dough and steamed buns were kept in a −20 °C refrigerator. Before SEM observation, the aliquots of dough and steamed-bun samples were immediately lyophilized in a freeze dryer. The dried samples were installed on a sample stage and fully sputtered with gold using a vacuum ion particle sprayer (JEC-3000FC, Japan Electronics Co., Ltd., Tokyo, Japan). The adopted sputtering conditions included a working sputtering pressure of 2.0 Pa, sputtering current of 30 mA, and sputtering time of 130 s. The samples were then mounted on the stage of a scanning electron microscope (JSM-IT 700HR, Japan Electronics Co., Ltd.) and photographed at a voltage of 25 kV, with 100 to 1200 times enlargement. The emission current in the observation chamber was 88 μA, and the distance between the sample and the lens was 10 mm.

### 4.10. Data Analysis

The data were statistically analyzed utilizing SPSS software (Version 17.0, SPSS Incorporated, [44]). For the control (CK) and PD- or PIF-added samples, one-way ANOVA and Duncan’s new multiple-range test were employed to compare multiple averages. Statistical significance was assessed at the *p* < 0.05 level. For examining the main effects of DF species and addition levels, univariate general linear model (UGLM) analysis was used; the LSD test was adopted for multiple comparisons to determine the significance of differences among the means.

## Figures and Tables

**Table 1:** Effect of adding DF on the specific volume of steamed buns.

Factor	Level	Volume (cm^3^)	Mass (g)	Specific Volume (cm^3^/g)
DF	PD	181.0 ± 2.0 ^d^	103.47 ± 0.13 ^c^	1.747 ± 0.019 ^e^
Species	PIF	198.0 ± 2.0 ^b^	102.89 ± 0.13 ^d^	1.921 ± 0.019 ^b^
Addition	0	160.0±3.2 ^e^	99.92±0.21 ^f^	1.601±0.030 ^f^
(%)	3	190.0±3.2 ^c^	101.51±0.21 ^e^	1.872±0.030 ^bc^
	5	190.0±3.2 ^c^	103.30±0.21 ^c^	1.840±0.030 ^cd^
	7	187.5±3.2 ^c^	104.84±0.21 ^b^	1.789±0.030 ^de^
	10	220.0±3.2 ^a^	106.35±0.21 ^a^	2.069±0.030 ^a^

Notes: PD, polydextrose; PIF, polydextrose + inulin + fructooligosaccharides. All data are expressed in the form of mean ± SD; number of repetitions—*n* = 3. Each DF species and each DF addition quantity have 15 and 6 values for UGLM analysis, respectively. Different superscript letters indicate significant differences (*p* < 0.05) within the column.

**Table 2:** Effect of adding DF on the color of steamed buns.

Factor	Level	L	−a*	b*	∆E
DF	PD	85.28 ± 0.26 ^a^	1.972 ± 0.015 ^c^	15.68 ± 0.10 ^b^	18.83 ± 0.17 ^c^
Species	PIF	82.80 ± 0.26 ^c^	2.067 ± 0.015 ^b^	15.04 ± 0.10 ^c^	20.04 ± 0.17 ^b^
Addition	0	82.09 ± 0.40 ^d^	2.040 ± 0.024 ^b^	15.49 ± 0.16 ^b^	20.85 ± 0.27 ^a^
(%)	3	83.01 ± 0.40 ^c^	1.927 ± 0.024 ^d^	14.86 ± 0.16 ^c^	19.76 ± 0.27 ^b^
	5	84.55 ± 0.40 ^b^	1.938 ± 0.024 ^cd^	14.89 ± 0.16 ^c^	18.73 ± 0.27 ^c^
	7	85.05 ± 0.40 ^ab^	2.082 ± 0.024 ^ab^	15.95 ± 0.16 ^a^	19.18 ± 0.27 ^c^
	10	85.51 ± 0.40 ^a^	2.112 ± 0.024 ^a^	15.62 ± 0.16 ^b^	18.67 ± 0.27 ^c^

Note: PD, polydextrose; PIF, polydextrose + inulin + fructooligosaccharides. L indicates brightness, −a* represents greenish tones, b* represents yellowish tones, and ΔE quantifies the color difference. All data are expressed in the form of mean ± SD; number of trial repetitions—*n* = 3. Each DF species and each DF addition amount have 15 and 6 values for UGLM analysis, respectively. Different superscript letters indicate significant differences (*p* < 0.05) in the column.

**Table 3:** Effect of added DF on the sensory attributes of steamed buns made with low-gluten flour.

Factor	Levels	Specific Volume	Width/Height Ratio	Elasticity	Surface Color	Surface Structure
DF	PD	9.40 ± 0.14 ^c^	3.00 ± 0.08 ^e^	8.23 ± 0.14 ^bc^	7.70 ± 0.16 ^b^	7.20 ± 0.19 ^b^
Species	PIF	10.00 ± 0.14 ^b^	3.40 ± 0.08 ^c^	8.28 ± 0.14 ^bc^	7.85 ± 0.16 ^b^	7.93 ± 0.19 ^a^
Addition	0	7.00 ± 0.23 ^e^	5.00 ± 0.13 ^a^	7.75 ± 0.23 ^d^	7.00 ± 0.25 ^c^	6.88 ± 0.30 ^b^
(%)	3	9.00 ± 0.23 ^d^	4.00 ± 0.13 ^b^	8.18 ± 0.23 ^bcd^	7.94 ± 0.25 ^ab^	8.19 ± 0.30 ^a^
	5	10.00 ± 0.23 ^b^	3.00 ± 0.13 ^d^	8.00 ± 0.23 ^cd^	7.81 ± 0.25 ^b^	6.81 ± 0.30 ^b^
	7	10.00 ± 0.23 ^b^	2.50 ± 0.13 ^f^	8.56 ± 0.23 ^ab^	7.81 ± 0.25 ^b^	7.75 ± 0.30 ^a^
	10	12.50 ± 0.23 ^a^	1.50 ± 0.13 ^g^	8.75 ± 0.23 ^a^	8.31 ± 0.25 ^a^	8.19 ± 0.30 ^a^
**Factor**	**Level**	**Internal structure**	**Toughness**	**Adhesiveness**	**Taste**	**Total score**
DF	PD	16.80 ± 0.18 ^b^	8.28 ± 0.15 ^bc^	8.18 ± 0.14 ^b^	4.60 ± 0.08 ^a^	73.38 ± 0.67 ^c^
Species	PIF	17.35 ± 0.18 ^a^	8.10 ± 0.15 ^c^	8.13 ± 0.14 ^b^	4.65 ± 0.08 ^a^	75.68 ± 0.67 ^b^
Addition	0	16.75 ± 0.29 ^b^	7.38 ± 0.24 ^d^	7.75 ± 0.22 ^c^	4.63 ± 0.12 ^a^	70.13 ± 1.07 ^d^
(%)	3	17.25 ± 0.29 ^ab^	8.06 ± 0.24 ^c^	7.75 ± 0.22 ^c^	4.56 ± 0.12 ^a^	74.94 ± 1.07 ^bc^
	5	17.13 ± 0.29 ^ab^	7.88 ± 0.24 ^c^	8.06 ± 0.22 ^bc^	4.56 ± 0.12 ^a^	73.25 ± 1.07 ^c^
	7	16.69 ± 0.29 ^b^	8.56 ± 0.24 ^b^	8.44 ± 0.22 ^a^	4.63 ± 0.12 ^a^	74.94 ± 1.07 ^bc^
	10	17.56 ± 0.29 ^a^	9.06 ± 0.24 ^a^	8.75 ± 0.22 ^a^	4.75 ± 0.12 ^a^	79.38 ± 1.07 ^a^

Notes: PD, polydextrose; PIF, polydextrose + inulin + fructooligosaccharides. All data are expressed in the form of mean ± SD; number of trial repetitions—*n* = 3. Each DF species and each DF addition amount have 15 and 6 values for UGLM analysis, respectively. Different superscript letters indicate significant differences (*p* < 0.05) in the column.

**Table 4:** Effect of adding DF on the texture parameters of steamed buns.

Factor	Level	Hardness (g)	Adhesive Force (g)	Adhesiveness (0.01 mJ)	Springiness (0.1 mm)	Cohesiveness (0.1)	Resilience	Gumminess (g)	Chewiness (mJ)
DF	PD	565.1 ± 16.6 ^cd^	3.27 ± 0.48 ^c^	2.33 ± 0.35 ^d^	4.31 ± 0.06 ^b^	7.39 ± 0.05 ^c^	10.78 ± 0.05 ^abc^	458.6 ± 11.3 ^b^	48.4 ± 1.2 ^b^
Species	PIF	603.3 ± 16.6 ^b^	4.79 ± 0.48 ^b^	4.20 ± 0.35 ^ab^	4.22 ± 0.06 ^b^	7.37 ± 0.05 ^c^	10.69 ± 0.05 ^bc^	446.2 ± 11.3 ^b^	46.8 ± 1.2 ^bc^
Addition	0	433.0 ± 26.2 ^e^	1.37 ± 0.75 ^d^	1.33 ± 0.55 ^e^	4.57 ± 0.09 ^a^	6.50 ± 0.07 ^d^	10.86 ± 0.08 ^a^	330.9 ± 17.9 ^d^	34.7 ± 1.9 ^d^
(%)	3	529.1 ± 26.2 ^d^	4.45 ± 0.75 ^b^	3.67 ± 0.55 ^bc^	4.28 ± 0.09 ^b^	7.73 ± 0.07 ^a^	10.83 ± 0.08 ^bc^	416.0 ± 17.9 ^c^	44.2 ± 1.9 ^c^
	5	691.6 ± 26.2 ^a^	6.18 ± 0.75 ^a^	5.00 ± 0.55 ^a^	4.25 ± 0.09 ^b^	7.67 ± 0.07 ^a^	10.67 ± 0.08 ^bc^	529.6 ± 17.9 ^a^	55.4 ± 1.9 ^a^
	7	669.5 ± 26.2 ^a^	4.23 ± 0.75 ^bc^	3.33 ± 0.55 ^bc^	4.32 ± 0.09 ^b^	7.58 ± 0.07 ^ab^	10.65 ± 0.08 ^c^	533.8 ± 17.9 ^a^	56.3 ± 1.9 ^a^
	10	597.8 ± 26.2 ^bc^	3.90 ± 0.75 ^bc^	3.00 ± 0.55 ^cd^	3.92 ± 0.09 ^c^	7.43 ± 0.07 ^bc^	10.65 ± 0.08 ^c^	451.9 ± 17.9 ^b^	47.2 ± 1.9 ^bc^

Note: PD, polydextrose; PIF, polydextrose + inulin + fructooligosaccharides. All data are expressed in the form of mean ± SD; number of trial repetitions—*n* = 3. Each DF species and each DF addition amount have 15 and 6 values for UGLM analysis, respectively. Different superscript letters indicate significant differences (*p* < 0.05) in the column.

**Table 5:** Effect of added DF on the pasting parameters of low-gluten wheat flour.

Factor	Level	Trough Viscosity (cP)	Peak Viscosity (cP)	Breakdown Viscosity (cP)	Final Viscosity (cP)	Setback Viscosity (cP)	Peak Time (min)	Pasting Temp. (°C)
DF	PD	1886 ± 7 ^c^	2782 ± 4 ^c^	896 ± 7 ^c^	3052 ± 3 ^c^	1165 ± 7 ^b^	6.308 ± 0.015 ^bc^	73.76 ± 0.10 ^c^
Species	PIF	1887 ± 7 ^c^	2761 ± 4 ^d^	874 ± 7 ^d^	3045 ± 3 ^d^	1158 ± 7 ^b^	6.335 ± 0.015 ^b^	73.98 ± 0.10 ^b^
Addition	0	2217 ± 11 ^a^	3225 ± 6 ^a^	1008 ± 10 ^a^	3454 ± 5 ^a^	1237 ± 11 ^a^	6.401 ± 0.023 ^a^	70.13 ± 0.15 ^f^
(%)	3	1969 ± 11 ^b^	2941 ± 6 ^b^	972 ± 10 ^b^	3191 ± 5 ^b^	1222 ± 11 ^a^	6.302 ± 0.023 ^b^	70.27 ± 0.15 ^f^
	5	1894 ± 11 ^c^	2772 ± 6 ^c^	878 ± 10 ^d^	3055 ± 5 ^c^	1161 ± 11 ^b^	6.325 ± 0.023 ^bc^	70.91 ± 0.15 ^e^
	7	1767 ± 11 ^d^	2595 ± 6 ^e^	829 ± 10 ^e^	2891 ± 5 ^e^	1124 ± 11 ^c^	6.290 ± 0.023 ^c^	71.78 ± 0.15 ^d^
	10	1588 ± 7 ^e^	2325 ± 6 ^f^	738 ± 10 ^f^	2651 ± 5 ^f^	1063 ± 11 ^d^	6.290 ± 0.023 ^c^	86.25 ± 0.15 ^a^

Notes: PD, polydextrose; PIF, polydextrose + inulin + fructooligosaccharides. All data are expressed as mean ± SD; number of trial repetitions—*n* = 3. Each DF species and each DF addition amount have 15 and 6 values for UGLM analysis, respectively. Different superscript letters indicate significant differences (*p* < 0.05) in the column.

**Table 6:** Effect of added DF on the thermal parameters of low-gluten wheat flour measured on day 0.

Factor	Level	Enthalpy (J/g)	*T*_o_ (°C)	*T*_p_ (°C)	*T*_c_ (°C)	Peak Width (°C)	Peak Height (0.01 mW/mg)
DF	PD	5.692 ± 0.031 ^b^	56.02 ± 0.06 ^bc^	62.59 ± 0.10 ^bc^	69.07 ± 0.17 ^ab^	3.93 ± 0.03 ^cd^	11.67 ± 0.12 ^ab^
Species	PIF	5.528 ± 0.031 ^c^	56.13 ± 0.06 ^b^	62.76 ± 0.10 ^bc^	69.30 ± 0.17 ^a^	3.97 ± 0.03 ^bcd^	11.31 ± 0.12 ^c^
Addition	0	5.364 ± 0.048 ^d^	55.87 ± 0.10 ^c^	61.97 ± 0.16 ^d^	68.80 ± 0.27 ^b^	3.73 ± 0.05 ^e^	11.41 ± 0.19 ^bc^
(%)	3	5.677 ± 0.048 ^b^	55.95 ± 0.10 ^c^	62.50 ± 0.16 ^c^	69.07 ± 0.27 ^ab^	3.88 ± 0.05 ^d^	11.56 ± 0.19 ^abc^
	5	6.004 ± 0.048 ^a^	55.93 ± 0.10 ^c^	62.85 ± 0.16 ^b^	69.40 ± 0.27 ^a^	4.03 ± 0.05 ^ab^	11.93 ± 0.19 ^a^
	7	5.761 ± 0.048 ^b^	56.05 ± 0.10 ^bc^	62.85 ± 0.16 ^b^	69.42 ± 0.27 ^a^	4.10 ± 0.05 ^a^	11.67 ± 0.19 ^ab^
	10	5.243 ± 0.048 ^e^	56.57 ± 0.10 ^a^	63.20 ± 0.16 ^a^	69.25 ± 0.27 ^ab^	4.00 ± 0.05 ^ab^	10.87 ± 0.19 ^d^

Notes: PD, polydextrose; PIF, polydextrose + inulin + fructooligosaccharides. All data are expressed in the form of mean ± SD; number of trial repetitions—*n* = 3. *T*_o_, onset temperature of gelatinization; *T*_p_, peak temperature of gelatinization; *T*_c_, conclusion temperature of gelatinization. Each DF species and each DF addition amount have 15 and 6 values for UGLM analysis, respectively. Different superscript letters indicate significant differences (*p* < 0.05) in the column.

**Table 7:** Effect of added DF on the thermal parameters of low-gluten wheat flour measured on day 21.

Factor	Level	Enthalpy (J/g)	*T*_o_ (°C)	*T*_p_ (°C)	*T*_c_ (°C)	Peak Width (°C)	Peak Height (0.01 mW/mg)	Aging (%)
DF	PD	0.782 ± 0.040 ^e^	48.28 ± 0.25 ^ab^	56.64 ± 0.11 ^b^	63.90 ± 0.28 ^b^	5.11 ± 0.13 ^c^	1.39 ± 0.06 ^e^	13.93 ± 0.77 ^e^
Species	PIF	1.188 ± 0.040 ^b^	47.46 ± 0.25 ^cd^	56.49 ± 0.11 ^b^	63.92 ± 0.28 ^b^	5.89 ± 0.13 ^a^	1.97 ± 0.06 ^b^	21.58 ± 0.77 ^b^
Addition	0	1.390 ± 0.064 ^a^	48.37 ± 0.39 ^ab^	59.20 ± 0.17 ^a^	63.27 ± 0.43 ^b^	5.63 ± 0.21 ^ab^	2.47 ± 0.10 ^a^	25.92 ± 1.21 ^a^
(%)	3	1.082 ± 0.064 ^c^	47.13 ± 0.39 ^d^	56.08 ± 0.17 ^c^	64.87 ± 0.43 ^a^	6.02 ± 0.21 ^a^	1.74 ± 0.10 ^c^	19.12 ± 1.21 ^c^
	5	0.926 ± 0.064 ^d^	47.97 ± 0.39 ^bc^	55.80 ± 0.17 ^cd^	64.05 ± 0.43 ^ab^	5.52 ± 0.21 ^b^	1.57 ± 0.10 ^cd^	15.65 ± 1.21 ^de^
	7	0.651 ± 0.064 ^f^	48.80 ± 0.39 ^a^	56.08 ± 0.17 ^c^	63.87 ± 0.43 ^b^	5.60 ± 0.21 ^ab^	1.12 ± 0.10 ^f^	11.36 ± 1.21 ^f^
	10	0.879 ± 0.064 ^de^	47.08 ± 0.39 ^d^	55.67 ± 0.17 ^d^	63.50 ± 0.43 ^b^	4.72 ± 0.21 ^d^	1.52 ± 0.10 ^de^	16.72 ± 1.21 ^cd^

Notes: PD, polydextrose; PIF, polydextrose + inulin + fructooligosaccharides. All data are expressed in the form of mean ± SD; number of trial repetitions—*n* = 3. *T*_o_, onset temperature of gelatinization; *T*_p_, peak temperature of gelatinization; *T*_c_*,* conclusion temperature of gelatinization. Each DF species and each DF addition amount have 15 and 6 values for UGLM analysis, respectively. Different superscript letters indicate significant differences (*p* < 0.05) in a column.

**Table 8:** Effect of added DF on the thermomechanical parameters of low-gluten wheat dough.

Factor	Level	DDT (min)	DST (min)	C1 (0.1 Nm)	C2 (0.1 Nm)	C3 (Nm)
DF	PD	8.989 ± 0.077 ^b^	8.213 ± 0.084 ^c^	6.55 ± 0.07 ^c^	2.78 ± 0.03 ^c^	1.905 ± 0.008 ^a^
Species	PIF	8.867 ± 0.077 ^bc^	8.217 ± 0.084 ^c^	6.60 ± 0.07 ^c^	2.70 ± 0.03 ^d^	1.829 ± 0.008 ^d^
Addition	0	8.483 ± 0.121 ^e^	10.000 ± 0.132 ^ab^	7.69 ± 0.11 ^a^	3.68 ± 0.05 ^a^	1.883 ± 0.012 ^b^
(%)	3	8.430 ± 0.121 ^e^	9.867 ± 0.132 ^b^	6.89 ± 0.11 ^b^	2.95 ± 0.05 ^b^	1.871 ± 0.012 ^bc^
	5	8.667 ± 0.121 ^de^	10.183 ± 0.132 ^a^	6.60 ± 0.11 ^c^	2.68 ± 0.05 ^d^	1.873 ± 0.012 ^b^
	7	8.757 ± 0.121 ^cd^	6.267 ± 0.132 ^d^	6.24 ± 0.11 ^d^	2.31 ± 0.05 ^e^	1.862 ± 0.012 ^bc^
	10	10.303 ± 0.121 ^a^	4.758 ± 0.132 ^e^	5.45 ± 0.11 ^e^	2.06 ± 0.05 ^f^	1.847 ± 0.012 ^cd^
**Factor**	**Level**	**C4 (Nm)**	**C5 (Nm)**	**C1–Cs (0.01 Nm)**	**C3–C4 ** ** (0.1 Nm)**	**C3/C4**
DF	PD	1.445 ± 0.009 ^d^	2.883 ± 0.016 ^b^	2.233 ± 0.169 ^b^	4.601 ± 0.135 ^bc^	1.327 ± 0.009 ^c^
Species	PIF	1.476 ± 0.009 ^c^	2.830 ± 0.016 ^cd^	1.907 ± 0.169 ^b^	3.530 ± 0.135 ^d^	1.255 ± 0.009 ^e^
Addition	0	1.272 ± 0.014 ^f^	2.788 ± 0.025 ^d^	1.200 ± 0.267 ^c^	6.110 ± 0.214 ^a^	1.481 ± 0.014 ^a^
(%)	3	1.382 ± 0.014 ^e^	2.832 ± 0.025 ^cd^	0.750 ± 0.267 ^c^	4.890 ± 0.214 ^b^	1.354 ± 0.014 ^b^
	5	1.445 ± 0.014 ^d^	2.846 ± 0.025 ^bc^	0.817 ± 0.267 ^c^	4.280 ± 0.214 ^c^	1.296 ± 0.014 ^d^
	7	1.527 ± 0.014 ^b^	2.821 ± 0.025 ^cd^	1.217 ± 0.267 ^c^	3.352 ± 0.214 ^d^	1.220 ± 0.014 ^f^
	10	1.678 ± 0.014 ^a^	2.998 ± 0.025 ^a^	6.367 ± 0.267 ^a^	1.697 ± 0.214 ^e^	1.104 ± 0.014 ^g^
**Factor**	**Level**	**C5–C4(Nm)**	**α ** ** (−0.01 Nm/min)**	**β ** ** (0.01 Nm/min)**	**γ ** ** (−0.01 Nm/min)**	** **
DF	PD	1.438 ± 0.018 ^b^	6.813 ± 0.183 ^b^	56.29 ± 1.05 ^b^	18.28 ± 2.24 ^b^	
Species	PIF	1.354 ± 0.018 ^cd^	7.280 ± 0.183 ^a^	50.89 ± 1.05 ^cd^	11.32 ± 2.24 ^d^	
Addition	0	1.516 ± 0.028 ^a^	6.867 ± 0.291 ^ab^	59.67 ± 1.66 ^a^	13.07 ± 3.53 ^bcd^	
(%)	3	1.450 ± 0.028 ^b^	7.233 ± 0.291 ^ab^	53.33 ± 1.66 ^c^	25.37 ± 3.53 ^a^	
	5	1.400 ± 0.028 ^bc^	7.233 ± 0.291 ^ab^	52.97 ± 1.66 ^cd^	18.70 ± 3.53 ^abc^	
	7	1.294 ± 0.028 ^e^	7.233 ± 0.291 ^ab^	52.17 ± 1.66 ^cd^	11.83 ± 3.53 ^cde^	
	10	1.320 ± 0.028 ^d^	6.667 ± 0.291 ^b^	49.83 ± 1.66 ^d^	5.03 ± 3.53 ^e^	

Notes: PD, polydextrose; PIF, polydextrose+ inulin + fructooligosaccharides. All data are expressed in the form of mean ± SD; number of trial repetitions―*n* = 3. DST, dough stability time; DDT, dough development time; C1, maximum consistency; Cs, 8-min torque; C2, minimum consistency; C3, maximum gelatinization torque; C4, persistent viscosity; C5, setback end torque; C1–Cs, protein weakness degree torque; C3–C4, starch breakdown torque; C3/C4, amylase activity; C5–C4, starch setback torque; β, gelatinization speed; α, speed of heating; γ, enzymatic degradation. Each DF species and each DF addition amount have 15 and 6 values for UGLM analysis, respectively. Different superscript letters indicate significant differences (*p* < 0.05) in the column.

**Table 9:** Effect of added DF on the starch structure of low-gluten wheat dough and steamed buns.

Food	Factor	Level	R_1022/995_	R_1047/1022_	R_1068/1022_
Dough	DF	PD	1.074 ± 0.003 ^a^	0.909 ± 0.003 ^c^	0.742 ± 0.008 ^c^
	Species	PIF	1.039 ± 0.003 ^c^	0.942 ± 0.003 ^a^	0.840 ± 0.008 ^a^
	Addition	0	1.050 ± 0.004 ^b^	0.937 ± 0.004 ^a^	0.827 ± 0.012 ^a^
	(%)	3	1.055 ± 0.004 ^b^	0.923 ± 0.004 ^b^	0.783 ± 0.012 ^b^
		5	1.055 ± 0.004 ^b^	0.925 ± 0.004 ^b^	0.787 ± 0.012 ^b^
		7	1.055 ± 0.004 ^b^	0.921 ± 0.004 ^b^	0.782 ± 0.012 ^b^
		10	1.067 ± 0.004 ^a^	0.920 ± 0.004 ^b^	0.778 ± 0.012 ^b^
Steamed	Factor	PD	1.097 ± 0.003 ^b^	0.904 ± 0.003 ^bc^	0.739 ± 0.003 ^c^
bread	DF	PIF	1.092 ± 0.003 ^b^	0.909 ± 0.003 ^b^	0.749 ± 0.003 ^b^
	Species	0	1.065 ± 0.004 ^c^	0.928 ± 0.002 ^a^	0.797 ± 0.005 ^a^
	Addition	3	1.105 ± 0.004 ^a^	0.900 ± 0.002 ^c^	0.726 ± 0.005 ^d^
	(%)	5	1.096 ± 0.004 ^b^	0.900 ± 0.002 ^c^	0.725 ± 0.005 ^d^
		7	1.096 ± 0.004 ^b^	0.904 ± 0.002 ^bc^	0.741 ± 0.005 ^bc^
		10	1.110 ± 0.004 ^a^	0.900 ± 0.002 ^c^	0.731 ± 0.005 ^cd^

Notes: PD, polydextrose; PIF, polydextrose + inulin + fructooligosaccharides. All data are expressed in the form of mean ± SD; number of trial repetitions—*n* = 3. Each DF species and each DF addition amount have 15 and 6 values for UGLM analysis, respectively. Different superscript letters indicate significant differences (*p* < 0.05) in the column.

**Table 10:** Effect of added DF on the protein secondary structure of low-gluten wheat dough and steamed buns.

Food	Factor	Level	β-Sheets (%)	Random Coils (%)	α-Helixes (%)	β-Turns (%)
Dough	DF	PD	50.929 ± 0.205 ^c^	16.268 ± 0.054 ^a^	16.876 ± 0.082 ^a^	15.927 ± 0.072 ^a^
	Species	PIF	53.298 ± 0.205 ^a^	15.605 ± 0.054 ^c^	15.907 ± 0.082 ^c^	15.200 ± 0.072 ^d^
	Addition	0	53.460 ± 0.325 ^a^	15.561 ± 0.085 ^c^	15.868 ± 0.129 ^c^	15.110 ± 0.114 ^d^
	(%)	3	51.531 ± 0.325 ^b^	16.011 ± 0.085 ^b^	16.615 ± 0.129 ^b^	15.843 ± 0.114 ^ab^
		5	51.685 ± 0.325 ^b^	16.089 ± 0.085 ^b^	16.561 ± 0.129 ^b^	15.666 ± 0.114 ^bc^
		7	51.894 ± 0.325 ^b^	15.985 ± 0.085 ^b^	16.472 ± 0.129 ^b^	15.649 ± 0.114 ^bc^
		10	51.974 ± 0.325 ^b^	16.038 ± 0.085 ^b^	16.440 ± 0.129 ^b^	15.548 ± 0.114 ^c^
Steamed	Factor	PD	52.124 ± 0.070 ^b^	15.995 ± 0.029 ^b^	16.360 ± 0.029 ^c^	15.521 ± 0.023 ^d^
bread	DF	PIF	52.225 ± 0.070 ^b^	15.924 ± 0.029 ^c^	16.338 ± 0.029 ^c^	15.513 ± 0.023 ^d^
	Species	0	54.045 ± 0.111 ^a^	15.378 ± 0.047 ^d^	15.655 ± 0.045 ^d^	14.912 ± 0.036 ^f^
	Addition	3	51.426 ± 0.111 ^c^	16.185 ± 0.047 ^a^	16.648 ± 0.045 ^b^	15.740 ± 0.036 ^b^
		5	51.059 ± 0.111 ^d^	16.235 ± 0.047 ^a^	16.758 ± 0.045 ^a^	15.948 ± 0.036 ^a^
		7	52.135 ± 0.111 ^b^	15.950 ± 0.047 ^c^	16.333 ± 0.045 ^c^	15.581 ± 0.036 ^c^
		10	52.198 ± 0.111 ^b^	16.048 ± 0.047 ^b^	16.350 ± 0.045 ^c^	15.404 ± 0.036 ^e^

Notes: PD, polydextrose; PIF, polydextrose + inulin + fructooligosaccharides. All data are expressed in the form of mean ± SD; number of trial repetitions—*n* = 3. Each DF species and each DF addition amount have 15 and 6 values for UGLM analysis, respectively. Different superscript letters indicate significant differences (*p* < 0.05) in the column.

## Data Availability

The original contributions presented in this study are included in this article; other inquiries can be directed to the corresponding author.

## Supplementary Materials

The following supporting information can be downloaded at: https://www.mdpi.com/article/10.3390/gels12090764/s1, Tables S1–S10, the raw data of the present study corresponding to Table 1, Table 2, Table 3, Table 4, Table 5, Table 6, Table 7, Table 8, Table 9 and Table 10; Figure S1. The pasting curves of DF-added low-gluten wheat flour; Figure S2. The 0-day and 21-day thermal property curves of DF-added low-gluten wheat flour; Figure S3. The FTIR spectra curves of wheat dough and steamed buns with DF added.

## References

[1] Allergies N., EFSA Panel on Dietetic Products (2010). Scientific opinion on dietary reference values for carbohydrates and dietary fibre. EFSA J..

[2] CDC, NCFHS (NCHS) (2005). National Health and Nutrition Examination Survey.

[3] Yang X.G., Yang Y.X., Ge K.Y. (2025). How to reshape the dietary logic of 1.4 billion people: The evolution of the dietary guidelines for Chinese residents and universal health practices. Sci. China.

[4] Gu L.L. (2022). “White Paper on Dietary Fiber Intake of Chinese Residents” Shows that 95% of Chinese Residents Lack Dietary Fiber. https://www.sohu.com/a/519198361_121086365.

[5] Mieszkowska A., Marzec A. (2016). Effect of polydextrose and inulin on texture and consumer preference of short-dough biscuits with chickpea flour. LWT—Food Sci. Technol..

[6] Parlan P., Nurhadi B., Ermawar R.A., Sikin A.M. (2026). Comparative study of physicochemical and thermal stability of different commercial soluble dietary fibres as alternative glass formers for spray-dried food powders. Int. J. Food Prop..

[7] Guanyan Research Report (2021). The Forecast of the Market Competition Pattern and Development Trend of China Polyglucose Industry on the Analysis Report of 2021 Year. https://market.chinabaogao.com/shipin/101455602R021.html.

[8] Li J.T., Wang M. (2020). Effect of composite powder of chickpea and pea on dough characteristics and the quality of steamed bread. Food Res. Dev..

[9] Zhao Y.J., Wang J.Y., Mou A.Q., Shen X., Pan Y.N., Li L. (2025). Study on the effect mechanism of highland barley Beta-glucan on deterioration of wheat dough with different gluten strengths. Grain Oils.

[10] Xie X.H., Hao M.Y., Fan Y.C., Zhang B., Wang N., Guo F.J. (2020). Effect of polydextrose on hydration and structure of gluten during frozen storage. Food Ferment. Ind..

[11] Guimarães D.J.S., Zambelli R.A., Afonso M.R.A., Pontes D.F. (2025). Cryoprotective potential of vegetable powders and polydextrose in frozen bread doughs. Int. J. Refrig..

[12] Jing Y., Wang W.X., Yang L.Z., Liu Y.Q., Shi M.M. (2020). Effect of resistant starch and polydextrose on the quality of steamed bread. Sci. Technol. Food Ind..

[13] Liu C., Dai B., Luo X.H., Song H.D., Li X.J. (2025). Polydextrose addition improves the chewiness and extended shelf-life of Chinese steamed bread through the formation of a sticky, elastic networkstructure. Gels.

[14] Xiong Z.W., Chen C.L., Wang C., Ren Y.R., Xie Y.J., Wang Q. (2018). Effects of inluin on farinograph and gel texture properties of flour with different gluten. Sci. Technol. Food Ind..

[15] (2014). Specific Wheat Flour. Issued Nov. 28th, 2024.

[16] Capriles V.D., Arêas J.A. (2013). Effects of prebiotic inulin-type fructans on structure, quality, sensory acceptance and glycemic response of gluten-free breads. Food Funct..

[17] Hosseininezhad M., Anvari H., Zhiani M., Abedfar A. (2017). Evaluating the effect of inulin supplementary on the sensory and textural properties of prebiotic bread (Taftoon). Res. Innov. Food Sci. Technol..

[18] Younes M., Aquilina G., Castle L., Engel K.H., Fowler P., Fürst P., Gürtler R., Gundert-Remy U., Husøy T. (2021). Re-evaluation of polydextrose (E 1200) as a food additive. EFSA J..

[19] (2026). The Difference Between Fructooligosaccharides and Polydextrose. https://www.mfk.com/article/7665004.shtml.

[20] (2026). The Difference Between Inulin and Fructooligosaccharides. http://www.zankeji.com/jingyan_962043/.

[21] Liu T., Duan H., Mao X., Yu X. (2020). Influence of flaxseed flour as a partial replacement for wheat flour on the characteristics of Chinese steamed bread. RSC Adv..

[22] Jiang Y. (2009). Preliminary evaluation of the application function of polydextrose in bread making. Tianjin Sci. Technol..

[23] GLMU, Lu W.D. (2002). SPSS for Windows Statistical Analysis, Version 2.

[24] Nopianti R., Huda N., Ismail N., Ariffin F., Easa A.M. (2013). Effect of polydextrose on physicochemical properties of threadfin bream (*Nemipteru* spp.) surimi during frozen storage. J. Food Sci. Technol..

[25] Ansari F., Pourjafar H., Pimentel T.C. (2021). Effect of inulin, polydextrose, or resistant starch on the quality parameters of prebiotic bread. Biointerface Res. Appl. Chem..

[26] Beltrão Martins R., Nunes M.C., M Ferreira L.M., A Peres J., RNA Barros A.I., Raymundo A. (2020). Impact of a corn flour on gluten-free dough rheology properties. Foods.

[27] Ye Y., Liao L., Yang R.X., Zhang S.J., Zhang J.Y., Zhang J.Y., Wu W.G., Zhang Y. (2024). Inhibitory effect of different degree of polymerization inulin on the retrogradation of rice starch gels and fresh rice noodles. LWT-Food Sci. Technol..

[28] Schirmer M., Jekle M., Arendt E., Becker T. (2012). Physicochemical interactions of polydextrose for sucrose replacement in pound cake. Food Res. Int..

[29] Chopin Application Laboratory (M.A. Handbook) (2009). Rheological and Enzymatic Analysis.

[30] Liaquat A., Ashraf H., Rehman H., Naveed S., Sadi H. (2026). Rheological characterization of composite wheat flours enriched with protein-rich plant sources for nutritional enhancement. Ital. J. Food Sci..

[31] Morris C., Morris G.A. (2012). The effect of inulin and fructo-oligosaccharide supplementation on the textural, rheological and sensory properties of bread and their role in weight management: A review. Food Chem..

[32] Ansari F., Pimentel T.C., Pourjafar H., Ibrahim S.A., Jafari S.M. (2022). The influence of prebiotics on wheat flour, dough, and bread properties; resistant starch, polydextrose, and inulin. Foods.

[33] Mohebbi Z., Homayouni A., Azizi M.H., Hosseini S.J. (2018). Effects of beta-glucan and resistant starch on wheat dough and prebiotic bread properties. J. Food Sci. Technol..

[34] Perez-Pirotto C., Moraga G., Hernando I., Cozzano S., Arcia P. (2022). Sorption isotherms, glass transition and bioactive compounds of ingredients enriched with soluble fibre from orange pomace. Foods.

[35] Wong K.Y., Thoo Y.Y., Tan C.P., Siow L.F. (2022). Moisture absorption behavior and thermal properties of sucrose replacer mixture containing inulin or polydextrose. Appl. Food Res..

[36] Feng Y., Ren Z.W. (2021). Effects of barley β-glucan on dough and gluten properties. Grain Oils.

[37] do Carmo M.M.R., Walker J.C.L., Novello D., Caselato V.M., Sgarbient V.C., Ouwehand A.C., Andreollo N.A., Hiane P.A., dos Santos E.F. (2016). Polydextrose: Physiological function and effects on health. Nutrients.

[38] (2018). Inspection of Grain And Oils—Quality Evaluation of Wheat Steamed Bread.

[39] Wang M.Y., Liu C., Luo X.H., Wu J.Z., Li X.J. (2025). Effect of polydextrose on the cooking and gelatinization properties and microstructure of Chinese early indica rice. Gels.

[40] (2007). Chinese Steamed Bread Made of Wheat Flour.

[41] (2010). Determination of Pasting Properties of Rice–Rapid Visco Analyzer Method.

[42] Ha M., Jeong H.Y., Lim S.T., Chung H.J. (2022). The cooking method features controlling eating quality of cooked rice: An explanation from the view of starch structure in leachate and morphological characteristics. Food Res. Int..

[43] Hang Y.Y., Zhang T.T., Qi D., Hu Y.Q. (2024). Effects and mechanism of polydextrose on the freezing-thawing stability of tilapia surimi. China Food Addit..

[44] SPSS Inc (2006). SPSS for Windows, Release 17.0.1.

