# Supplementary material for: Effect of Polydextrose and Its Complex on the Pasting, Function, and Microstructural Characteristics of Low-Gluten Wheat Dough and Steamed Buns
Source: Gels. 2026 Aug 26;12(9):764. doi: 10.3390/gels12090764 (PMC13606658; doi:10.3390/gels12090764)
Supplement: Supplementary file 1 [file gels-12-00764-s001.zip › gels-4397170-supplementary.pdf]

Supplementary Materials

Effect of polydextrose and its complex on the pasting, function and microstructural characteristics of low-gluten wheat dough and steamed bun

Shiyu Chang[1], Ruijun Chen[1,2], Bing Dai[1]and Xingjun Li[1]*

[1]Academy of National Food and Strategic Reserves Administration, National Engineering Research Center for Grain Storage and Transportation, Beijing 102209, China;csy@ags.ac.cn (S.C);bingdai3@126.com (B.D.);[2]College of Food Science and Engineering, Wuhan Polytechnic University, Wuhan 430048, China; ruijunchen1@126.com(R.C);

* Correspondence: lxj@ags.ac.cn

Table S1. Effect of adding DFs on the specific volume of steamed buns

DF	Addition Amount (%)	Volume (cm^3)	Mass (g)	Specific volume (cm^3/g)
PD	0	160±0[d]	99.92±0.77[e]	1.601±0.012[d]
	3	180±0[c]	102.07±0.10[d]	1.763±0.002[b]
	5	180±0[c]	103.73±0.26[c]	1.735±0.004[c]
	7	185±0[b]	105.28±0.31[b]	1.757±0.005[b]
	10	200±0[a]	106.39±0.23[a]	1.879±0.004[a]
PIF	0	160±0[d]	99.92±0.77[e]	1.601±0.012[e]
	3	200±0[b]	100.94±0.40[d]	1.981±0.008[b]
	5	200±0[b]	102.86±0.67[c]	1.944±0.013[c]
	7	190±0[c]	104.40±0.21[b]	1.819±0.004[d]
	10	240±0[a]	106.30±0.64[a]	2.258±0.014[a]

Notes: DF, dietary fiber; PD, polydextrose; PIF, polydextrose + inulin + fructooligosaccharides. All data are expressed as mean ± SD; number of trial repetitions—n=3. The different superscript letters in the column indicate the significant differences ($p< 0.05$) for each DF species.

Table S2. Effect of adding DFs on the colour of steamed buns.

DF	Addition Amount (%)	L	—a	b	△E
PD	0	82.09±0.58[e]	2.040±0.010[a]	15.49±0.17[b]	20.85±0.32[a]
	3	83.54±0.33[d]	1.907±0.081[bc]	15.54±0.24[ab]	19.83±0.12[b]
	5	86.27±0.20[c]	1.910±0.026[c]	15.32±0.13[b]	17.83±0.22[c]
	7	86.89±0.07[b]	1.993±0.046[ab]	16.05±0.31[a]	18.04±0.23[c]
	10	87.60±0.42[a]	2.010±0.036[ab]	16.02±0.24[a]	17.62±0.39[c]
PIF	0	82.09±0.58[b]	2.040±0.010[b]	15.49±0.17[a]	20.85±0.32[a]
	3	82.48±0.46[b]	1.947±0.059[c]	14.18±0.19[c]	19.69±0.42[b]
	5	82.82±0.54[ab]	1.967±0.047[c]	14.47±0.22[bc]	19.63±0.27[b]
	7	83.20±0.21[a]	2.170±0.044[a]	15.86±0.49[a]	20.31±0.49[b]
	10	83.42±0.20[a]	2.213±0.025[a]	15.22±0.57[ab]	19.72±0.50[b]

Notes: DF, dietary fiber; PD, polydextrose; PIF, polydextrose + inulin + fructooligosaccharides. All data are expressed as mean ± SD; number of trial repetitions—n=3. The different superscript letters in the column indicate the significant differences ($p < 0.05$) for each DF species.

Table S3. Effect of added DFs on the sensory attributes of steamed bread made from weak gluten flour.

DF	Addition Amount (%)	Specific volume	Width/height ratio	Elasticity	Surface color	Surface structure
PD	0	7±0^{d}	5±0^{a}	7.75±0.89^{a}	7.00±0.93^{b}	6.88±0.99^{a}
	3	10±0^{b}	3±0^{b}	8.13±0.35^{a}	7.88±1.13^{ab}	7.88±0.99^{a}
	5	9±0^{c}	3±0^{b}	8.25±0.89^{a}	8.00±0.00^{a}	7.00±0.53^{a}
	7	10±0^{b}	3±0^{b}	8.50±0.93^{a}	7.75±1.04^{ab}	6.88±1.73^{a}
	10	11±0^{a}	1±0^{c}	8.50±0.93^{a}	7.88±0.35^{ab}	7.38±1.06^{a}
PIF	0	7±0^{e}	5±0^{a}	7.75±0.89^{a}	7.00±0.93^{b}	6.88±0.99^{b}
	3	8±0^{d}	5±0^{a}	8.25±0.71^{a}	8.00±1.60^{ab}	8.50±1.41^{ab}
	5	11±0^{b}	3±0^{b}	7.75±1.16^{a}	7.63±1.19^{ab}	6.63±1.51^{b}
	7	10±0^{c}	2±0^{c}	8.63±1.19^{a}	7.88±0.99^{ab}	8.63±1.19^{ab}
	10	14±0^{a}	2±0^{c}	9.00±0.93^{a}	8.75±0.71^{a}	9.00±0.76^{a}

DF	Addition Amount (%)	Internal structure	Toughness	Adhesiveness	Taste	Total score
PD	0	16.75±1.75^{a}	7.38±0.92^{a}	7.75±0.89^{a}	4.63±0.52^{a}	70.13±4.76^{a}
	3	17.50±0.53^{a}	8.38±1.19^{a}	7.63±1.06^{a}	4.63±0.52^{a}	75.00±4.07^{a}
	5	16.75±0.46^{a}	8.00±1.31^{a}	8.13±1.25^{a}	4.63±0.52^{a}	72.75±4.03^{a}
	7	15.88±1.46^{a}	8.63±0.92^{a}	8.63±0.74^{a}	4.50±0.53^{a}	73.75±3.01^{a}
	10	17.13±0.64^{a}	9.00±0.93^{a}	8.75±0.71^{a}	4.63±0.52^{a}	75.25±1.67^{a}
PIF	0	16.75±1.75^{a}	7.38±0.92^{b}	7.75±0.89^{a}	4.63±0.52^{a}	70.13±4.76^{b}
	3	17.00±1.07^{a}	7.75±1.04^{ab}	7.88±0.83^{a}	4.50±0.53^{a}	74.88±4.55^{b}
	5	17.50±0.93^{a}	7.75±0.89^{a}	8.00±0.76^{a}	4.50±0.53^{a}	73.75±4.33^{b}
	7	17.50±0.93^{a}	8.50±0.93^{ab}	8.25±0.71^{a}	4.75±0.46^{a}	76.13±4.82^{ab}
	10	18.00±0.76^{a}	9.13±0.64^{a}	8.75±0.89^{a}	4.88±0.35^{a}	83.50±3.42^{a}

Notes: DF, dietary fiber; PD, polydextrose; PIF, polydextrose + inulin + fructooligosaccharides. All data are expressed as mean ± SD; number of trial repetitions—n=8. The different superscript letters in the column indicate the significant differences ($p< 0.05$) for each DF species.

Table S4. Effect of adding DFs on the texture profiles of weak-gluten steamed buns

DF	Addition Amount (%)	Hardness (g)	Adhesive Force(g)	Adhesiveness (0.01mJ)	Springiness (0.1mm)	Cohesiveness (0.1)	Resilience	Gumminess (g)	Chewiness (mJ)
PD	0	433.0±30.1^{b}	1.37±0.72^{c}	1.33±0.58^{b}	4.57±0.12^{a}	6.50±0.10^{b}	10.86±0.06^{a}	330.9±10.9^{c}	34.7±1.5^{c}
	3	576.3±92.1^{a}	5.90±0.17^{a}	3.67±0.58^{a}	4.33±0.21^{a}	7.60±0.17^{a}	10.77±0.26^{ab}	436.3±62.9^{b}	46.1±7.1^{b}
	5	629.5±36.1^{a}	5.01±0.46^{b}	3.33±0.58^{a}	4.33±0.31^{a}	7.77±0.21^{a}	10.67±0.06^{b}	482.7±1.8^{b}	50.5±0.5^{b}
	7	615.5±61.4^{a}	2.01±0.89^{c}	1.33±1.15^{b}	4.43±0.15^{a}	7.60±0.36^{a}	10.95±0.09^{a}	571.9±34.0^{a}	61.4±3.9^{a}
	10	571.3±43.2^{a}	2.07±0.45^{c}	2.00±1.00^{b}	3.90±0.10^{b}	7.50±0.17^{a}	10.63±0.11^{b}	471.2±21.7^{b}	49.1±1.9^{b}
PIF	0	433.0±30.1^{c}	1.37±0.72^{c}	1.33±0.58^{c}	4.57±0.12^{a}	6.50±0.10^{d}	10.86±0.06^{a}	330.9±10.9^{d}	34.7±1.5^{e}
	3	481.9±43.1^{c}	3.01±0.80^{b}	3.67±0.58^{b}	4.23±0.21^{b}	7.87±0.06^{a}	10.89±0.08^{a}	395.7±4.81^{c}	42.3±0.5^{d}
	5	753.7±17.8^{a}	7.37±1.59^{a}	6.67±1.53^{a}	4.17±0.06^{b}	7.57±0.06^{b}	10.66±0.21^{ab}	576.6±19.6^{a}	60.3±2.9^{a}
	7	723.6±63.7^{a}	6.47±2.25^{a}	5.33±2.08^{ab}	4.20±0.56^{ab}	7.57±0.15^{bc}	10.35±0.24^{b}	495.5±59.5^{b}	51.3±3.3^{b}
	10	624.3±47.9^{b}	5.73±1.86^{a}	4.00±1.00^{b}	3.93±0.15^{b}	7.37±0.12^{c}	10.67±0.22^{ab}	432.5±21.7^{b}	45.2±1.4^{c}

Notes: DF, dietary fiber; PD, polydextrose; PIF, polydextrose + inulin + fructooligosaccharides. All data are expressed as mean ± SD; number of trial repetitions—n=3. The different superscript letters in the column indicate the significant differences ($p < 0.05$) for each DF species.

Table S5. Effect of added DFs on the pasting parameters of weak-gluten wheat flours

DF	Addition Amount (%)	Trough viscosity (cP)	Peak viscosity (cP)	Breakdown viscosity (cP)	Final viscosity (cP)	Setback viscosity (cP)	Peak time (min)	Pasting temp. (°C)
PD	0	2217±39^{a}	3225±14^{a}	1008±37^{a}	3454±15^{a}	1237±40^{ab}	6.40±0.07^{a}	70.13±0.06^{c}
	3	1963±15^{b}	2945±12^{b}	982±3^{a}	3199±10^{b}	1237±6^{a}	6.27±0.01^{b}	70.12±0.06^{c}
	5	1878±18^{c}	2782±3^{c}	904±18b	3059±2^{c}	1181±16^{b}	6.27±0.01^{b}	70.67±0.45^{b}
	7	1786±43^{d}	2617±6^{d}	831±38^{c}	2896±16^{d}	1109±32^{c}	6.31±0.03^{ab}	71.78±0.83^{b}
	10	1587±2^{e}	2339±14^{e}	753±13^{d}	2649±14^{e}	1062±14^{d}	6.29±0.03^{b}	86.08±0.46^{a}
PIF	0	2217±39^{a}	3225±14^{a}	1008±37^{a}	3454±15^{a}	1237±40^{a}	6.40±0.07^{a}	70.13±0.06^{d}
	3	1975±12^{b}	2937±9^{b}	962±16^{a}	3182±5^{b}	1207±15^{a}	6.33±0.07^{ab}	70.42±0.42^{cd}
	5	1909±34^{c}	2761±22^{c}	851±39^{b}	3052±11^{c}	1142±36^{b}	6.38±0.11^{ab}	71.15±0.48^{bc}
	7	1747±12^{d}	2573±9^{d}	826±3^{b}	2885±13^{d}	1139±1^{b}	6.27±0.01^{b}	71.78±0.03^{b}
	10	1588±22^{e}	2311±13^{e}	723±11^{c}	2653±5^{e}	1065±18^{c}	6.29±0.03^{b}	86.42±0.03^{a}

Notes: DF, dietary fiber; PD, polydextrose; PIF, polydextrose + inulin + fructooligosaccharides. All data are expressed as mean ± SD; number of trial repetitions—n=3. The different superscript letters in the column indicate the significant differences ($p < 0.05$) for each DF species.

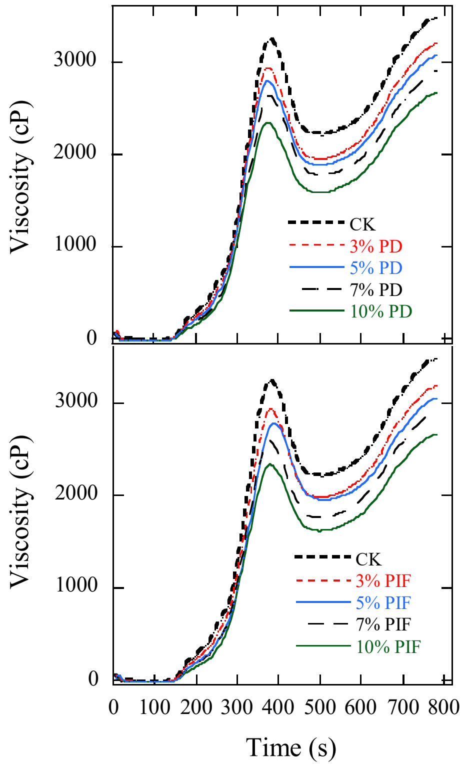

Figure S1. The pasting curves of DF added low-gluten wheat flour.

Table S6. Effect of added DFs on the thermal parameters of low-gluten wheat flours on day 0

DF	Addition Amount (%)	Enthalpy (J/g)	T_o (°C)	T_p (°C)	T_c (°C)	Peak width (°C)	Peak height (0.01mW/mg)
PD	0	5.364±0.063^{d}	55.87±0.06^{b}	61.97±0.06^{c}	68.80±0.70^{a}	3.73±0.06^{c}	11.41±0.12^{bc}
	3	5.758±0.038^{c}	55.90±0.17^{b}	62.30±0.10^{b}	68.97±0.61^{a}	3.87±0.06^{b}	11.73±0.68^{abc}
	5	6.241±0.041^{a}	55.77±0.15^{b}	62.73±0.06^{a}	69.10±0.26^{a}	4.00±0.10^{ab}	12.34±0.25^{a}
	7	5.861±0.059^{b}	56.00±0.36^{b}	62.73±0.42^{ab}	69.30±0.66^{a}	4.07±0.12^{a}	11.96±0.49^{ab}
	10	5.238±0.147^{d}	56.57±0.12^{a}	63.20±0.44^{a}	69.20±0.30^{a}	4.00±0.10^{ab}	10.89±0.48^{c}
PIF	0	5.364±0.063^{c}	55.87±0.06^{c}	61.97±0.06^{c}	68.80±0.70^{a}	3.73±0.06^{c}	11.41±0.12^{a}
	3	5.596±0.082^{b}	56.00±0.17^{bc}	62.70±0.17^{b}	69.17±0.31^{a}	3.90±0.10^{b}	11.38±0.27^{a}
	5	5.768±0.053^{a}	56.10±0.52^{abc}	62.97±1.15^{ab}	69.70±1.73^{a}	4.07±0.38^{abc}	11.52±1.00^{ab}
	7	5.661±0.048^{b}	56.10±0.10^{b}	62.97±0.12^{ab}	69.53±0.21^{a}	4.13±0.06^{a}	11.37±0.18^{a}
	10	5.249±0.061^{c}	56.57±0.31^{a}	63.20±0.30^{a}	69.30±0.10^{a}	4.00±0.01^{b}	10.85±0.25^{b}

Notes: DF, dietary fiber; PD, polydextrose; PIF, polydextrose + inulin + fructooligosaccharides. All data are expressed as mean ± SD; number of trial repetitions—n=3. The different superscript letters in the column indicate the significant differences ($p < 0.05$) for each DF species.

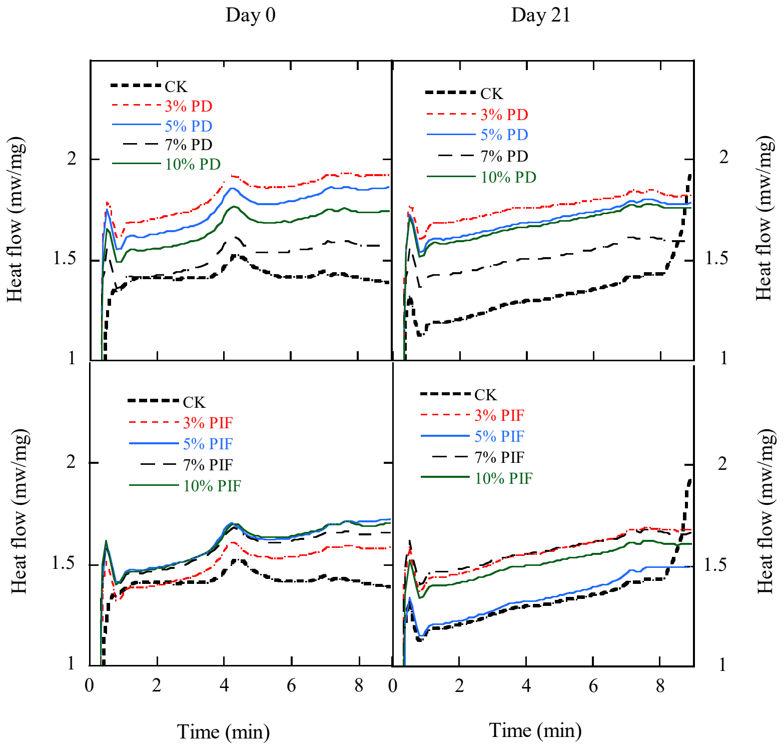

Figure S2. The 0-day and 21-day thermal property curves of DF added low-gluten wheat flour.

Table S7. Effect of added DFs on the thermal parameters of low-gluten wheat flours on day 21

DF	Addition Amount (%)	Enthalpy (J/g)	T_o (°C)	T_p (°C)	T_c (°C)	Peak width (°C)	Peak height (0.01mW/mg)	Aging (%)
PD	0	1.390±0.101^{a}	48.37±0.51^{ab}	59.20±0.01^{a}	63.27±0.15^{b}	5.63±0.06^{a}	2.47±0.11^{a}	25.92±2.12^{a}
	3	0.924±0.033^{b}	47.70±0.40^{b}	56.20±0.17^{b}	65.27±0.38^{a}	5.67±0.64^{ab}	1.58±0.05^{b}	16.04±0.62^{b}
	5	0.615±0.067^{c}	48.27±0.49^{ab}	55.87±0.21^{b}	62.87±0.99^{b}	5.23±1.11^{abc}	1.10±0.19^{c}	9.86±1.10^{c}
	7	0.450±0.016^{d}	48.63±0.06^{a}	56.27±0.98^{b}	65.01±0.36^{a}	4.90±0.17^{b}	0.78±0.01^{d}	7.67±0.25^{d}
	10	0.534±0.108^{cd}	48.43±1.12^{ab}	55.67±0.40^{b}	63.10±0.87^{b}	4.10±0.26^{c}	1.04±0.14^{c}	10.17±1.80^{c}
PIF	0	1.390±0.101^{a}	48.37±0.51^{a}	59.20±0.01^{a}	63.27±0.15^{b}	5.63±0.06^{c}	2.47±0.11^{a}	25.92±2.12^{a}
	3	1.241±0.134^{ab}	46.57±0.25^{b}	55.97±0.76^{b}	64.47±1.16^{ab}	6.37±0.57^{ab}	1.89±0.25^{b}	22.20±2.73^{ab}
	5	1.237±0.072^{ab}	47.67±1.89^{ab}	55.73±0.23^{b}	65.23±0.67^{a}	5.80±0.17^{bc}	2.03±0.09^{b}	21.45±1.18^{b}
	7	0.852±0.092^{c}	48.97±0.38^{a}	55.90±0.35^{b}	62.73±0.85^{b}	6.30±0.10^{a}	1.46±0.08^{c}	15.05±1.71^{c}
	10	1.221±0.033^{b}	45.73±0.93^{b}	55.67±0.06^{b}	63.90±0.10^{b}	5.33±0.23^{d}	2.01±0.05^{b}	23.27±0.90^{ab}

Notes: DF, dietary fiber; PD, polydextrose; PIF, polydextrose + inulin + fructooligosaccharides. All data

are expressed as mean ± SD; number of trial repetitions—n=3. The different superscript letters in the column indicate the significant differences ($p < 0.05$) for each DF species.

Table S8. Effect of added DFs on the thermomechanical parameters of low-gluten wheat dough

DF	Addition Amount (%)	DDT (min)	DST (min)	C1 (0.1Nm)	C2 (0.1Nm)	C3 (Nm)
PD	0	8.48±0.27^{bc}	10.00±0.10^{ab}	7.69±0.19^{a}	3.68±0.13^{a}	1.883±0.007^{b}
	3	8.62±0.07^{b}	9.83±0.12^{b}	7.02±0.10^{b}	3.06±0.06^{b}	1.904±0.007^{a}
	5	8.87±0.32^{b}	10.40±0.40^{a}	6.54±0.27^{c}	2.67±0.15^{c}	1.902±0.012^{ab}
	7	8.64±0.07^{b}	6.13±0.45^{c}	6.17±0.19^{c}	2.38±0.16^{cd}	1.921±0.017^{a}
	10	10.33±0.16^{a}	4.70±0.20^{d}	5.31±0.41^{d}	2.09±0.14^{d}	1.915±0.027^{ab}
PIF	0	8.48±0.27^{bc}	10.00±0.10^{a}	7.69±0.19^{a}	3.68±0.13^{a}	1.883±0.007^{a}
	3	8.24±0.13^{c}	9.90±0.46^{a}	6.76±0.18^{b}	2.83±0.10^{b}	1.837±0.007^{b}
	5	8.47±0.34^{bc}	9.97±0.15^{a}	6.66±0.08^{b}	2.69±0.05^{b}	1.844±0.010^{b}
	7	8.87±0.27^{b}	6.40±0.53^{b}	6.31±0.05^{c}	2.25±0.06^{c}	1.803±0.017^{c}
	10	10.27±0.59^{a}	4.82±0.33^{c}	5.59±0.58^{d}	2.03±0.13^{d}	1.779±0.017^{c}

DF	Addition Amount (%)	C4 (Nm)	C5 (Nm)	C1-Cs (0.01Nm)	C3-C4 (0.1Nm)	C3/C4
PD	0	1.272±0.017^{e}	2.788±0.080^{c}	1.200±0.557^{bc}	6.110±0.195^{a}	1.481±0.021^{a}
	3	1.381±0.030^{d}	2.824±0.079^{bc}	0.867±0.321^{bc}	5.230±0.261^{b}	1.379±0.027^{b}
	5	1.441±0.020^{c}	2.905±0.014^{b}	0.867±0.115^{c}	4.607±0.090^{c}	1.320±0.011^{c}
	7	1.511±0.017^{b}	2.861±0.076^{bc}	1.367±0.208^{b}	4.097±0.050^{d}	1.271±0.005^{d}
	10	1.619±0.047^{a}	3.038±0.081^{a}	6.867±0.737^{a}	2.963±0.202^{e}	1.183±0.018^{e}
PIF	0	1.272±0.017^{e}	2.788±0.080^{bc}	1.200±0.557^{bc}	6.110±0.195^{a}	1.481±0.021^{a}
	3	1.382±0.024^{d}	2.840±0.043^{b}	0.633±0.153^{b}	4.550±0.211^{b}	1.329±0.021^{b}
	5	1.449±0.016^{c}	2.786±0.009^{c}	0.767±0.231^{b}	3.953±0.211^{c}	1.273±0.017^{c}
	7	1.542±0.026^{b}	2.781±0.010^{c}	1.067±0.416^{b}	2.607±0.302^{d}	1.169±0.022^{d}
	10	1.736±0.040^{a}	2.957±0.020^{a}	5.867±1.762^{a}	0.430±0.262^{e}	1.025±0.016^{e}

DF	Addition Amount (%)	C5-C4(Nm)	α (-0.01Nm/min)	β (0.01 Nm/min)	γ (-0.01 Nm/min)
PD	0	1.516±0.080^{a}	6.867±0.416^{a}	59.67±2.58^{a}	13.07±17.67^{ab}
	3	1.443±0.069^{ab}	7.200±0.529^{a}	53.73±2.66^{b}	31.80±5.50^{a}
	5	1.464±0.018^{a}	7.267±1.301^{a}	54.87±2.61^{ab}	24.00±2.95^{a}
	7	1.349±0.061^{ab}	6.600±0.800^{a}	58.47±1.758^{a}	14.47±4.69^{b}
	10	1.419±0.085^{ab}	6.133±0.808^{a}	54.73±3.03^{ab}	8.07±2.72^{b}
PIF	0	1.516±0.080^{a}	6.867±0.416^{a}	59.67±2.58^{a}	13.07±17.67^{ab}
	3	1.457±0.065^{a}	7.267±0.115^{a}	52.93±2.23^{b}	18.93±11.32^{ab}
	5	1.337±0.010^{b}	7.200±0.400^{a}	51.07±3.52^{bc}	13.40±1.59^{a}
	7	1.238±0.025^{c}	7.867±0.702^{a}	45.87±6.40^{bc}	9.20±0.35^{b}
	10	1.221±0.024^{c}	7.200±0.721^{a}	44.93±2.73^{c}	2.00±0.53^{c}

Notes: DF, dietary fiber; PD, polydextrose; PIF, polydextrose + inulin + fructooligosaccharides. All data are expressed as mean ± SD; number of trial repetitions—n=3. The different superscript letters in the column indicate the significant differences ($p < 0.05$) for each DF species.

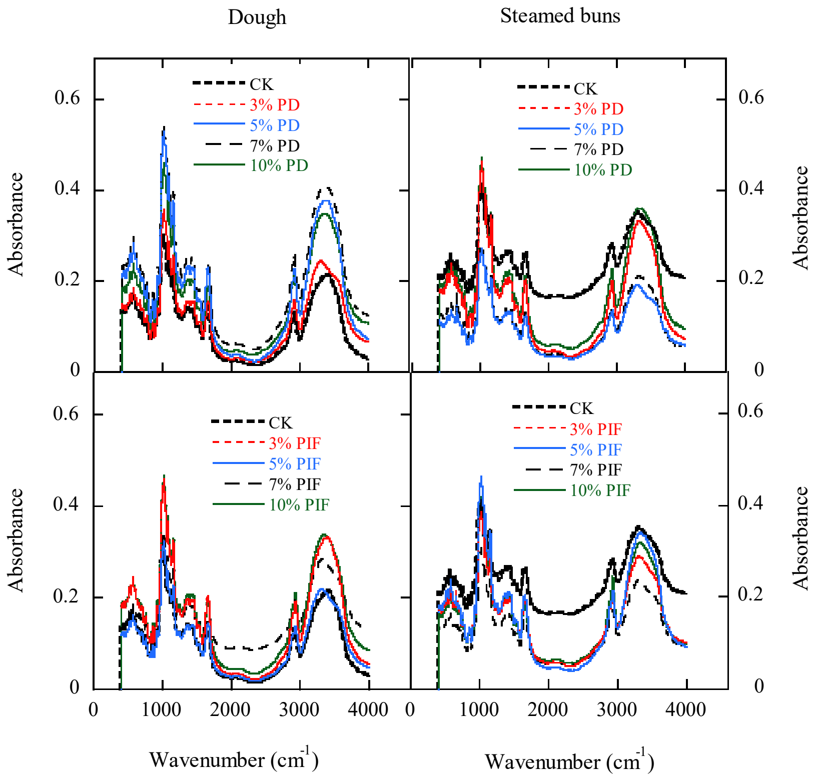

Figure S3. The FTIR spectra curves of wheat dough and steamed buns with DF added.

Table S9. Effect of added DFs on the starch structure of low-gluten wheat dough and steamed buns

Food	DF	Addition Amount (%)	$R_{1022/995}$	$R_{1047/1022}$	$R_{1068/1022}$
Dough	PD	0	1.0501±0.0019^{d}	0.9373±0.0014^{a}	0.8271±0.0037^{a}
		3	1.0785±0.0004^{b}	0.8947±0.0001^{d}	0.7043±0.0004^{d}
		5	1.0758±0.0011^{c}	0.9028±0.0014^{c}	0.7220±0.0035^{c}
		7	1.0749±0.0006^{c}	0.9056±0.0002^{b}	0.7314±0.0012^{b}
		10	1.0908±0.0010^{a}	0.9036±0.0001^{c}	0.7270±0.0007^{c}
	PIF	0	1.0501±0.0019^{a}	0.9373±0.0014^{b}	0.8271±0.0037^{b}
		3	1.0312±0.0002^{d}	0.9507±0.0043^{a}	0.8613±0.0100^{a}
		5	1.0335±0.0006^{cd}	0.9466±0.0026^{a}	0.8517±0.0038^{a}
		7	1.0352±0.0012^{c}	0.9374±0.0011^{b}	0.8321±0.0014^{b}
		10	1.0427±0.0010^{b}	0.9359±0.0028^{b}	0.8283±0.0054^{b}
Steamed buns	PD	0	1.0649±0.0018^{e}	0.9277±0.0081^{a}	0.7968±0.0167^{a}
		3	1.1251±0.0006^{a}	0.8918±0.0008^{d}	0.7068±0.0015^{d}
		5	1.0893±0.0005^{d}	0.9016±0.0004^{b}	0.7327±0.0007^{b}
		7	1.0966±0.0038^{c}	0.9011±0.0008^{b}	0.7315±0.0019^{b}
		10	1.1076±0.0014^{b}	0.8972±0.0009^{c}	0.7255±0.0030^{c}
	PIF	0	1.0649±0.0018^{e}	0.9277±0.0081^{a}	0.7968±0.0167^{a}
		3	1.0857±0.0023^{d}	0.9079±0.0016^{b}	0.7442±0.0045^{bc}
		5	1.1018±0.0013^{b}	0.8976±0.0006^{d}	0.7171±0.0016^{d}
		7	1.0956±0.0022^{c}	0.9074±0.0014^{b}	0.7501±0.0035^{b}
		10	1.1127±0.0012^{a}	0.9036±0.0016^{c}	0.7372±0.0033^{c}

Notes: DF, dietary fiber; PD, polydextrose; PIF, polydextrose + inulin + fructooligosaccharides. All data are expressed as mean ± SD; number of trial repetitions—n=3. The different superscript letters in the column indicate the significant differences ($p < 0.05$) for each DF species.

Table S10. Effect of added DFs on the protein secondary structure of low-gluten wheat dough and steamed buns

Food	DF	Addition Amount (%)	Beta-sheet (%)	Random coils (%)	Alpha-helixes (%)	Bea-turn (%)
Dough	PD	0	53.460±0.151[a]	15.561±0.048[c]	15.868±0.032[d]	15.109±0.073[e]
		3	49.400±0.030[d]	16.545±0.003[a]	17.468±0.012[a]	16.587±0.017[d]
		5	50.144±0.222[c]	16.522±0.069[a]	17.191±0.081[b]	16.142±0.072[b]
		7	50.736±0.176[b]	16.351±0.042[b]	16.959±0.067[c]	15.955±0.067[c]
		10	50.906±0.040[b]	16.362±0.020[b]	16.892±0.014[c]	15.839±0.010[d]
	PIF	0	53.460±0.151[ab]	15.561±0.048[bc]	15.868±0.032[b]	15.109±0.073[c]
		3	53.662±0.319[a]	15.477±0.128[bc]	15.761±0.083[b]	15.099±0.111[c]
		5	53.225±0.011[bc]	15.655±0.030[a]	15.929±0.016[a]	15.190±0.008[c]
		7	53.052±0.053[d]	15.619±0.015[ab]	15.985±0.053[a]	15.344±0.012[a]
		10	53.042±0.196[c]	15.714±0.073[c]	15.987±0.056[a]	15.256±0.068[b]
Steamed buns	PD	0	54.055±0.398[a]	15.378±0.125[d]	15.654±0.130[c]	14.912±0.144[d]
		3	51.105±0.174[c]	16.345±0.043[a]	16.787±0.080[a]	15.763±0.052[b]
		5	51.156±0.108[c]	16.137±0.023[b]	16.700±0.049[a]	16.005±0.036[a]
		7	52.250±0.165[b]	15.976±0.027[c]	16.262±0.079[b]	15.510±0.113[c]
		10	52.052±0.197[b]	16.137±0.066[b]	16.395±0.067[b]	15.415±0.065[c]
	PIF	0	54.055±0.398[a]	15.378±0.125[d]	15.654±0.130[e]	14.912±0.144[d]
		3	51.746±0.114[d]	16.026±0.025[b]	16.509±0.043[b]	15.717±0.046[b]
		5	50.961±0.096[e]	16.333±0.031[a]	16.814±0.035[a]	15.891±0.035[a]
		7	52.020±0.127[c]	15.924±0.035[c]	16.403±0.047[c]	15.651±0.046[b]
		10	52.344±0.121[b]	15.959±0.055[b]	16.305±0.040[d]	15.391±0.039[c]

Notes: DF, dietary fiber; PD, polydextrose; PIF, polydextrose + inulin + fructooligosaccharides. All data are expressed as mean ± SD; number of trial repetitions—n=3. The different superscript letters in the column indicate the significant differences ($p < 0.05$) for each DF species.
